# New Quinazolin-4(3H)-One Derivatives Incorporating Isoxazole Moiety as Antioxidant Agents: Synthesis, Structural Characterization, and Theoretical DFT Mechanistic Study

**DOI:** 10.3390/ph17101390

**Published:** 2024-10-18

**Authors:** Yassine Rhazi, Riham Sghyar, Noemi Deak, Bouchra Es-Sounni, Bouchra Rossafi, Albert Soran, Mustapha Laghmari, Azize Arzine, Asmae Nakkabi, Khalil Hammani, Samir Chtita, Mohammed M. Alanazi, Gabriela Nemes, Mohamed El. Yazidi

**Affiliations:** 1 Engineering Laboratory of Organometallic, Materials and Environment, Faculty of Sciences Dhar EL Mahraz, Sidi Mohamed Ben Abdellah University, P.O. Box 1796, Atlas, Fez 30000, Morocco; arzineaziz@gmail.com; 2 Faculty of Chemistry and Chemical Engineering, Babes-Bolyai University, 11 Arany Janos, 400028 Cluj-Napoca, Romania; noemi.deak@ubbcluj.ro (N.D.); gabriela.nemes@ubbcluj.ro (G.N.); 3 Laboratory of Applied Organic Chemistry, Faculty of Science and Techniques, Sidi Mohamed Ben Abdellah University, Routed ‘Imouzzer, P.O. Box 2202, Fez 30050, Morocco; riham.sghyar@usmba.ac.ma; 4 Laboratory of Innovative Materials and Biotechnologies of Natural Resources, Faculty of Sciences, Moulay Ismail University, P.O. Box 11201, Meknes 50000, Morocco; bouchrasounni@gmail.com; 5 Laboratory of Analytical and Molecular Chemistry, Faculty of Sciences Ben M’Sik, Hassan II University of Casablanca, P.O. Box 7955, Casablanca 20023, Morocco; bouchrarossafi7@gmail.com (B.R.); samirchtita@gmail.com (S.C.); 6 Supramolecular Organic and Organometallic Chemistry Centre, Chemistry Department, Faculty of Chemistry and Chemical Engineering, Babes-Bolyai University, 11 Arany Janos, 400028 Cluj-Napoca, Romania; asoran@chem.ubbcluj.ro; 7 Laboratory of Natural Resources and Environment, Polydisciplinary Faculty of Taza, Sidi Mohamed Ben Abdellah University of Fez, P.O. Box 1223, Taza-Gare, Taza 30050, Morocco; mustapha.laghmari@usmba.ac.ma (M.L.); khalil.hammani@usmba.ac.ma (K.H.); 8 Laboratory of Materials Engineering for the Environment and Natural Resources, Faculty of Sciences and Techniques, University of Moulay Ismail of Meknes, P.O. Box 509, Boutalamine, Errachidia 52000, Morocco; asmaenakkabi@yahoo.fr; 9 Department of Pharmaceutical Chemistry, College of Pharmacy, King Saud University, Riyadh 11451, Saudi Arabia; mmalanazi@ksu.edu.sa

**Keywords:** quinazolin-(3H)-one, isoxazole, DFT mechanistic study, antioxidant agents

## Abstract

**Background**: This research centers on the development and spectroscopic characterization of new quinazolin-4(3H)-one-isoxazole derivatives (**5a–e**). The aim was to investigate the regioselectivity of the 1,3-dipolar cycloaddition involving arylnitriloxides and N-propargylquinazolin-4(3H)-one, and to assess the antioxidant properties of the synthesized compounds. The synthetic approach started with the alkylation of quinazolin-4(3H)-one using propargyl bromide, followed by a 1,3-dipolar cycloaddition reaction. **Methods**: The structural identification of the products was performed using various spectroscopic methods, such as IR, 1H, 13C, and HMBC NMR, HRMS, and single-crystal X-ray diffraction. To further examine the regioselectivity of the cycloaddition, Density Functional Theory (DFT) calculations at the B3LYP/6-31G(d) level were employed. Additionally, the antioxidant potential of the compounds was tested in vitro using DPPH (2,2-Diphenyl-1-picrylhydrazyl)radical scavenging assays. The reaction selectively produced 3,5-disubstituted isoxazoles, with the regiochemical outcome being independent of the substituents on the phenyl ring. **Results**: Theoretical calculations using DFT were in agreement with the experimental results, revealing activation energies of −81.15 kcal/mol for P-1 and −77.32 kcal/mol for P-2, favoring the formation of P-1. An analysis of the Intrinsic Reaction Coordinate (IRC) confirmed that the reaction proceeded via a concerted but asynchronous mechanism. The antioxidant tests demonstrated that the synthesized compounds exhibited significant radical scavenging activity, as shown in the DPPH assay. The 1,3-dipolar cycloaddition of arylnitriloxides with N-propargylquinazolin-4(3H)-one successfully resulted in novel 3,5-disubstituted isoxazoles. **Conclusions**: The experimental findings were well-supported by theoretical predictions, and the antioxidant assays revealed strong activity, indicating the potential for future biological applications of these compounds.

## 1. Introduction

The body’s natural antioxidant defense system plays a vital role in maintaining physiological functions and protecting against the harmful effects of reactive oxygen species (ROS) and other oxidants. Excessive ROS are linked to the development of various oxidative stress-related diseases, including cardiovascular disorders [1], Alzheimer’s disease [2] and cancer [3]. Consequently, antioxidants are crucial in both the prevention and treatment of these conditions. Recent studies suggest that single-target drugs are increasingly prone to resistance, leading to the diminished efficacy of many promising drug candidates. It is now widely recognized that drugs designed to act on multiple targets or various sites within the same target tend to be more effective than those focused on a single target [4,5,6,7,8]. 

In light of this observation, the design of new, more effective antioxidant agents has become of great importance. Over the past decade, particular attention has been paid to the synthesis and discovery of more efficient antioxidant agents. A variety of heterocyclic compounds, including heteroatoms such as nitrogen, sulfur, and oxygen, have been explored [9,10]. 

**Figure 1:**
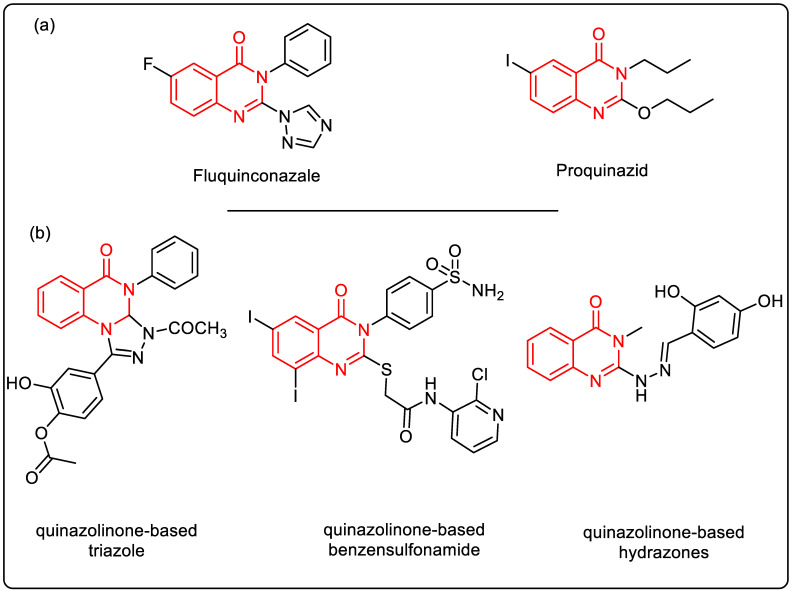
(**a**) Chemical structures of commercial drugs containing the quinazolinone backbone; (**b**) representative examples of diverse quinazolin4(3H)-ones with antioxidant activity. The quinazoline nucleus in red indicates its presence in drug structures and as an antioxidant.

Quinazolin-4-one is a structurally important motif found in a variety of synthetic derivatives with pharmaceutical purposes and in numerous natural alkaloids. In fact, it is an essential scaffold found in about 150 natural alkaloids and drugs [11]. The quinazolin-4-one skeleton is also crucial in the prevention and treatment of agricultural diseases, as seen in commercial fungicides such as proquinazid and fluquinconazole [12,13] (Figure 1a).

Compounds containing the quinazolin-4-one backbone are versatile molecules with diverse pharmacological activities, making it a promising candidate for the development of various drugs and therapeutic agents [14,15]. Such compounds have garnered significant interest from researchers because of their antioxidant properties [16,17,18,19,20] (Figure 1b) and the simplicity of their synthesis.

**Figure 2:**
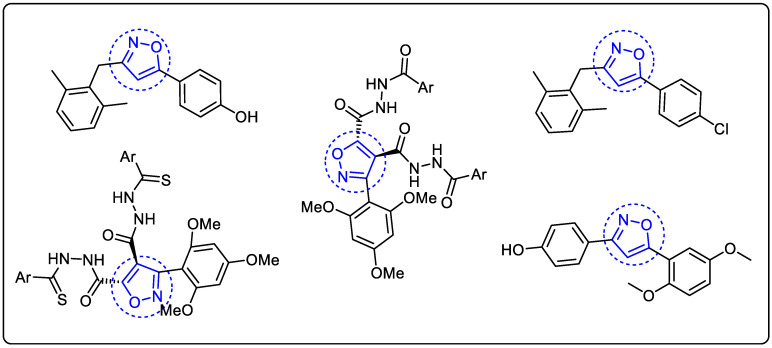
Isoxazole ring-based molecules with antioxidant activity. The isoxazole core in blue indicates its presence in the antioxidant agent structures.

Besides the quinazolin-4-one motif, the isoxazole ring is recognized as one of the most important heterocycles for the discovery of new agents in medicinal chemistry. Several derivatives of the isoxazole ring serve as basic structures for many drugs, including immunomodulatory [21,22], antimicrobial [23], antiviral [24], anticancer [22], antiplatelet, antithrombotic, triglyceride suppressing [25], antidiabetic [26], analgesic [27], and anti-Alzheimer [28]. They are also used in the production of pesticides and insecticides [29,30]. Some examples of isoxazole ring-based molecules with antioxidant activity are shown in Figure 2 [31,32,33].

To achieve the expected objectives, molecular hybridization technique is emerging as a new alternative [34,35,36]. This strategy involves the fusion of two or more distinct pharmacophores to create a new hybrid molecule that combines the properties of each pharmacophore. Currently, this strategy is widely used in the development of new drugs targeting various targets. It helps to minimize the risks associated with multiple side effects, which are characteristic of traditional treatments [37,38].

In the present work, we provide an overview of a series of quinazolin-4-one-isoxazole hybrids synthesized via N-alkylation and 1,3-dipolar cycloaddition reactions. A DFT study was conducted to rationalize the experimental findings and to gain deeper insight into the regioselectivity of the 1,3-dipolar cycloaddition between propargylated quinazolin-4(3H)-one and nitrile oxides, using the B3LYP/6-31G(d) computational level. Furthermore, the antioxidant properties of the synthesized compounds were assessed in vitro using the DPPH radical scavenging assay.

## 2. Results and Discussion

### 2.1. Synthesis and Spectral Analysis

**Scheme 1:**
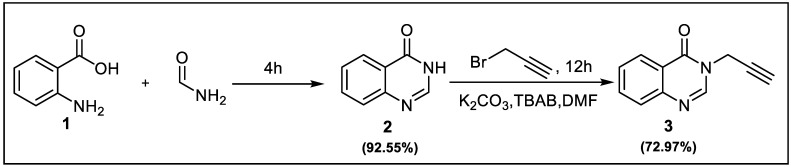
Synthesis of the dipolarophile **3**.

3-(prop-2-yn-1-yl)quinazoline-4(3H)-one **3**, used as a dipolarophile in this work, was prepared according to the strategy described in Scheme 1. Quinazoline-4(3H)-one **2** was prepared according to the procedure described in the literature from anthranilic acid (**1**) and formamide [39]. It was then alkylated with propargyl bromide under phase transfer catalysis (PTC) in the presence of tetra-n-butylammonium bromide (TBAB) [40]. 

The quinazolinone-isoxazole hybrids **5a–e** were prepared by the 1,3-dipolar cycloaddition reaction of N-propargyl quinazoline-4(3H)-one **3** and suitably substituted nitrile oxides. The nitrile oxides were generated in situ by the action of triethylamine as a base on their hydroxamoyl chloride precursors **4a–e** [41].

**Scheme 2:**
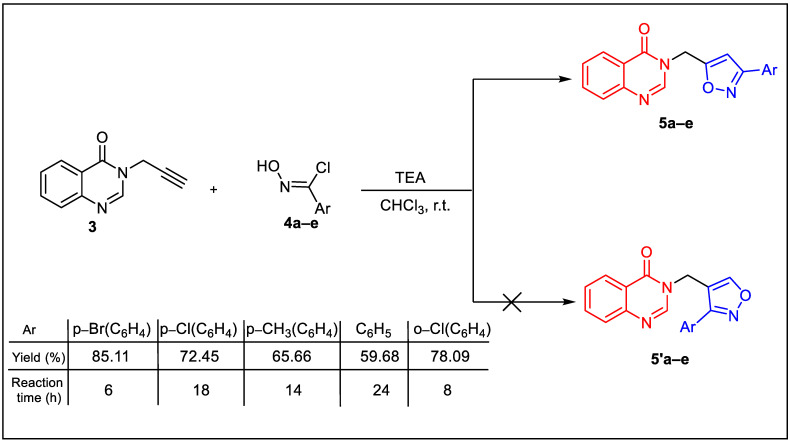
Synthesis procedure of the novel hybrid molecules (**5**). The quinazolinone core in red and the isoxazole core in blue to visualize their assembly within the same molecular fragment.

**Scheme 3:**
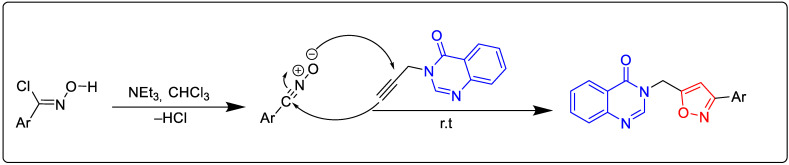
Possible reaction mechanism for the synthesis of new isoxazoles. The quinazolinone core in red and the isoxazole core in blue to visualize their assembly within the same molecular fragment.

The 1,3-cycloaddition reactions of N-propargyl quinazoline-4(3H)-one **3** were performed in chloroform at room temperature for 6 to 24 h, depending on the dipole used, yielding the 3,5-disubstituted compounds (**5**) as the sole regioisomers, with good yields and no traces of the (**5′**) regioisomers (Scheme 2). The observed regioselectivity aligns well with both NMR results and literature reports [32,33], which may indicate that the possible reaction mechanism for the 1,3-dipolar reaction between dipolarophile 3 and the nitrile oxide derivatives **4a–h** is as presented in Scheme 3.

The structures of the cycloadducts were confirmed through IR spectroscopy, ^1^H and ^13^C NMR, 2D HMBC NMR, HRMS, and single-crystal X-ray diffraction. The physical properties and spectroscopic data for compounds **5a–e**, while detailed spectral information can be found in the Supplementary Information.

High-resolution mass spectrometry (HRMS) confirmed the exact masses of the [M + H]^+^ ions for the synthesized hybrid compounds, supporting the proposed molecular structures. For instance, compound **5a** exhibited a molecular ion peak at m/z 383.021, matching the calculated mass for the molecular formula C_18_H_13_N_3_O_2_Br [M + H]. The spectrum also shows the presence of two isotopic peaks [M + H]^+^ at 382.018 and [M + H]^+2^ at 384.016 with approximatively the same intensity (100.00% and 98.10%), attesting the presence of the bromine atom. These findings are further supported by IR spectroscopy. The IR spectrum of compound **5a** displays a strong absorption band at 1672 cm^−^^1^, corresponding to the stretching vibration of the C=O bond in the quinazolinone carbonyl group. Additionally, bands at 1159 cm^−^^1^ and 1612 cm^−^^1^ are consistent with the stretching vibrations of the C–O and C=N bonds within the isoxazole ring, respectively.

The ^1^H and ^13^C NMR spectra of the hybrid compounds **5a–e** were recorded in DMSO-d_6_ or CDCl_3_, based on the solubility of each compound. The ^1^H NMR spectra display singlet peaks between 5.35 and 5.45 ppm, corresponding to the two protons of the methylene group (N–H_2_), and singlets within the 6.67–7.10 ppm range, which can be attributed to the methine protons (CH) in the isoxazole ring. Additionally, singlet signals observed between 7.57 and 8.59 ppm can be assigned to the methine protons (N=CH) of the quinazolinone ring.

In the ^13^C NMR spectra, signals for the quaternary carbons of the isoxazole ring are noted between 165.25 and 169.12 ppm for C_3_, and between 161.10 and 162.87 ppm for C_5_. Signals for the isoxazole carbons within the C–H group are found around 100 ppm. The carbonyl and imine carbons of the quinazolinone ring have corresponding signals at approximately 160 ppm and 148 ppm, respectively, while the aromatic carbons are found between 120 and 146.99 ppm.

The structural assignment of the **5a–e** hybrids is consistent with the literature data concerning the compounds obtained through 1,3-dipolar cycloaddition of arylnitriloxides to terminal alkynes [42]. According to the literature, only one 3,5-disubstituted regioisomer was obtained. This structure was characterized by the presence of a single singlet signal in the ^1^H NMR spectrum between 6.16–6.85 ppm assigned to the C_4_ carbon proton, and a signal around 100 ppm for the C_4_ carbon in the ^13^C NMR spectrum. In the case of the 3,4-disubstituted regioisomer, one would expect to observe highly deshielded signals for the C_5_ carbon atom and the corresponding proton bonded to this carbon atom [42,43].

**Figure 3:**
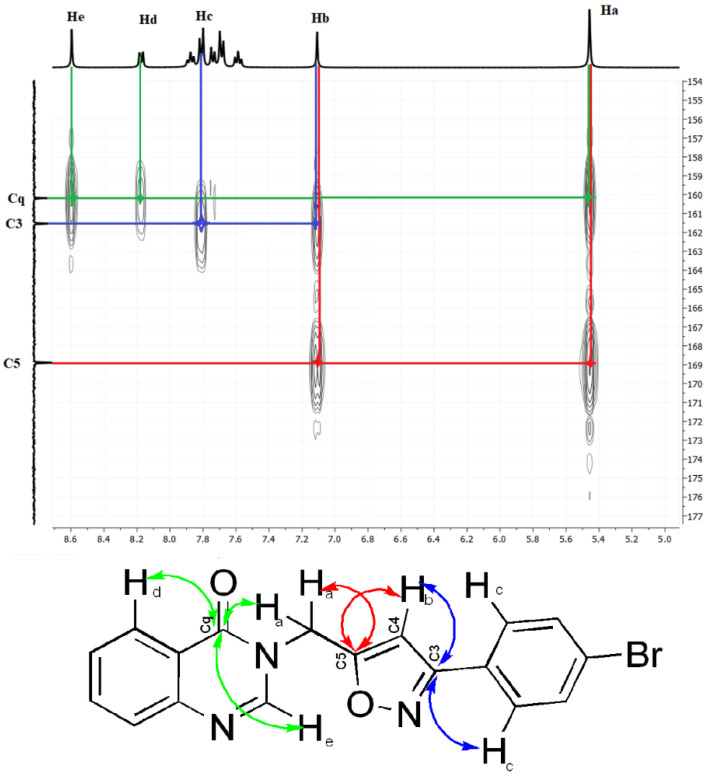
HMBC (^1^H-^13^C) spectrum of compound **5a**.

To confirm that the synthesized cycloadducts are indeed 3,5-disubstituted regioisomers, we used HMBC 2D NMR spectroscopy. The HMBC spectra enable the detection of C–H interactions over three bonds. To demonstrate how this technique can distinguish between 3,5- and 3,4-disubstituted isomers, an HMBC analysis was performed for compound **5a** and the important assignments are shown in Figure 3.

It is noteworthy that the signal in the ^13^C NMR at 169.1 ppm, corresponding to C5, correlates with two signals in the ^1^H NMR, one corresponding to a proton in the methine group (Hb), at 7.1 ppm, with ^2^J_CH_ coupling, the other to a proton in the methylene group (Ha), at 5.45 ppm, with ^2^J_CH_ coupling. Subsequently, the carbon atom at 161.8 ppm is correlated with two protons: one resonating at 7.85 ppm, representing the aromatic proton Hc (^3^J_CH_ coupling), and the other signal at 7.1 ppm, corresponding to a proton in the methine group (Hb), showing a ^2^J_CH_ coupling. These observations confirm that the signal at 161.8 ppm is for carbon atom C3 of the isoxazole ring. Finally, the carbon atom at 160.4 ppm is correlated with three signals at 8.59 ppm, 8.19 ppm, and 5.45 ppm, respectively, corresponding to the methine group (He) of the quinazolinone ring (^3^J_CH_ coupling), the aromatic proton (Hd) of the same ring (^3^J_CH_ coupling), and the methylene group (Ha) that connects the two nuclei (^3^J_CH_ coupling). These confirm that compound **5a** is the 3,5-disubstituted regioisomer and not the 3,4-disubstituted regioisomer.

### 2.2. X-Ray Diffraction Data and Crystal Structures of the Two Compounds ***5a*** and ***5c***

**Figure 4:**
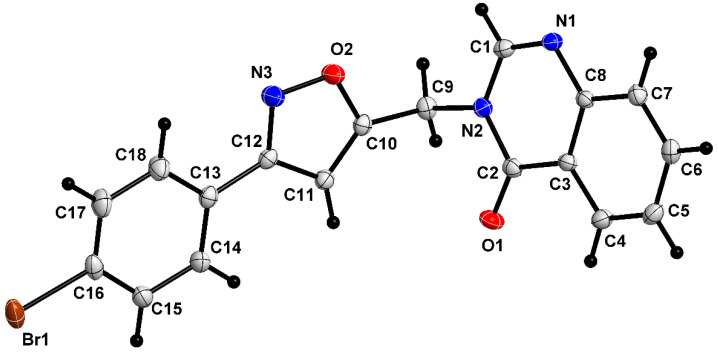
The asymmetric unit of **5a**, shown with 50% probability ellipsoids. Grey indicates carbon atom, Black indicates hydrogen atom, Red indicates Oxygen atom, and Blue indicates Nitrogen atom.

Compound **5a** crystallizes in the monoclinic *Pc* space group, with two molecules per unit cell. The asymmetric unit (Figure 4) shows the expected quinazoline and isoxazole moieties linked through a methylene bridge. 

**Figure 5:**
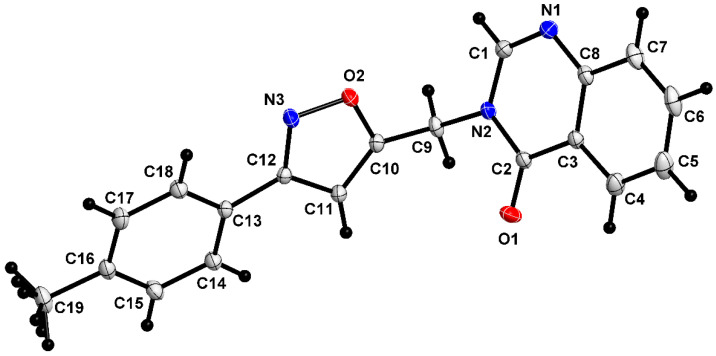
The asymmetric unit of **5c**, shown with 50% probability ellipsoids. Grey indicates the carbon atom, Black indicates the hydrogen atom, Red indicates the Oxygen atom, and Blue indicates the Nitrogen atom.

Compound **5c** crystallizes in the orthorhombic *Pbca* space group, with eight molecules per unit cell. The asymmetric unit of **5c** (Figure 5) is similar to that of **5a**. Table 1 presents the crystallographic data, experimental details of the data collection and structure refinements.

### 2.3. Antioxidant Activity

Given the significant damage inflicted by reactive oxygen species (ROS) and reactive nitrogen species (RNS) in the human body, it is crucial to identify new therapeutic agents that offer improved efficacy compared to current natural and synthetic antioxidants.

**Figure 6:**
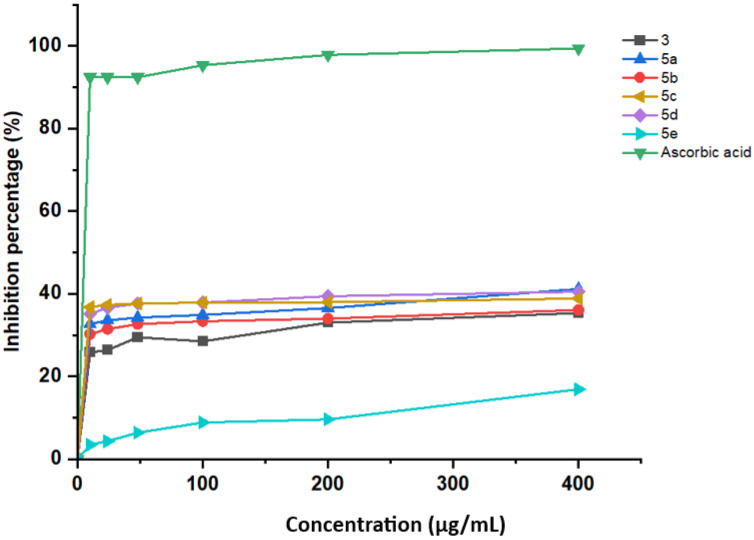
DPPH radical scavenging activity of products and ascorbic acid.

Quinazolin-4(3H)-ones and isoxazole are known for their large spectrum of biological activities; combining the two active pharmacophores may enhance the biological activity, in particular the antioxidant activity. In general, quinazolinone and isoxazole are not considered to possess significant antioxidant activity against free radicals such as DPPH, since they are relatively stable and do not contain functional groups capable of readily surrendering electrons or hydrogen atoms to reduce a free radical [44,45]. In this study, all synthesized hybrid compounds have shown moderate anti-DPPH activity (Figure 6), but these activities are lower than that of the positive control. Indeed, at a concentration of 400 µg/mL, the products inhibit DPPH radicals with a percentage which varies between 16.88 and 41.16%. These findings indicate that the IC_50_ values are greater than 400 µg/mL.

### 2.4. DFT Study

#### 2.4.1. Analysis Based on the Global Reactivity Indexes

To understand the mechanism and predict the reactivity of the 1,3-dipolar cycloaddition reaction under investigation, global indices such as chemical potential (μ), chemical hardness (η), electrophilicity (ω), and nucleophilicity (N) were calculated and are summarized in Table 2.

According to the data in Table 2, it can be observed that the electronic chemical potential of compound **3** (µ = −3.76 eV) is higher than that of compound **4d** (µ = −3.83 eV), this suggesting that during the 1,3-dipolar cycloaddition reaction, charge transfer is likely to occur from compound **3** to compound **4e**. Additionally, the nucleophilicity and electrophilicity indices for compound **3** are 2.80 eV and 2.77 eV, respectively, whereas those for compound **4e** are 2.78 eV and 2.91 eV. Thus, both reagents are categorized as moderate nucleophiles and strong electrophiles, within the electrophilicity and nucleophilicity scales [46,47]. Since these reagents display similar characteristics, and considering the earlier discussion about charge transfer from compound **3** to compound **4e**, we can infer that in this reaction, compound **3** acts as a nucleophile while compound **4e** functions as an electrophile.

**Figure 7:**
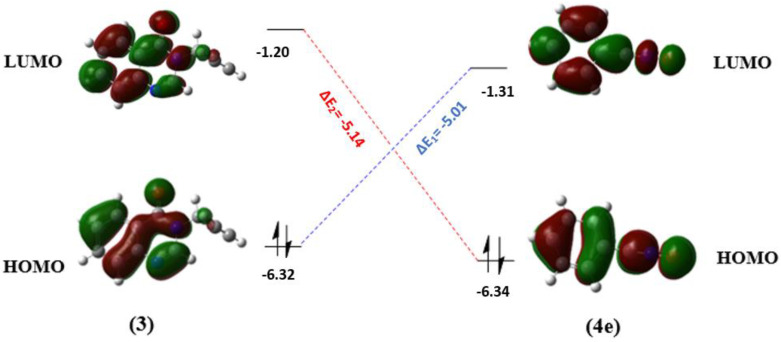
Interactions between the frontier molecular orbitals of **3** and **4e** involved in the 1,3-dipolar cycloaddition reaction, from a quantum calculation by DFT/6-31G(d) (energy gaps ΔE in eV).

The difference in energy between the HOMO orbital of compound **3** and the LUMO orbital of **4e** (ΔE_1_) is on the order of 5.01 eV, lower than that between the HOMO orbital of **4e** and the LUMO orbital of compound **3** (ΔE_2_) which is on the order of 5.14 eV (Figure 7), indicating that this reaction can be classified as an inverse-electron demand (IED) 1,3-dipolar cycloaddition reaction, and it is controlled by charge transfer; this transfer will occur from compound **3** to **4e**, confirming the results described earlier. 

#### 2.4.2. Energetic Study

**Scheme 4:**
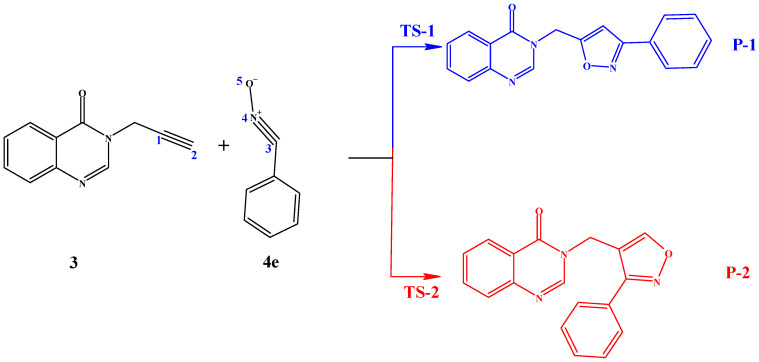
Regioisomeric reactive pathways associated with the 1,3-dipolar cycloaddition reaction between (**3**) and (**4e**). 1 = C_1_, 2 = C_2_, 3 = C_3_, 4 = N_4_, 5 = O_5_; Transition state-1 in blue, and transition state-2 in red.

Because of the asymmetry of both reactants, the 1,3-dipolar cycloaddition reaction between compounds **3** and **4e** can occur via two competitive reactive pathways, corresponding to two regioisomeric approach modes, P-1 involving the formation of single bonds C_1_–O_5_ and C_2_–C_3_, and P-2 implicating the formation of single bonds C_1_–C_3_ and C_2_–O_5_. This occurs through two distinct transition states, TS-1 and TS-2, as shown in Scheme 4. 

**Figure 8:**
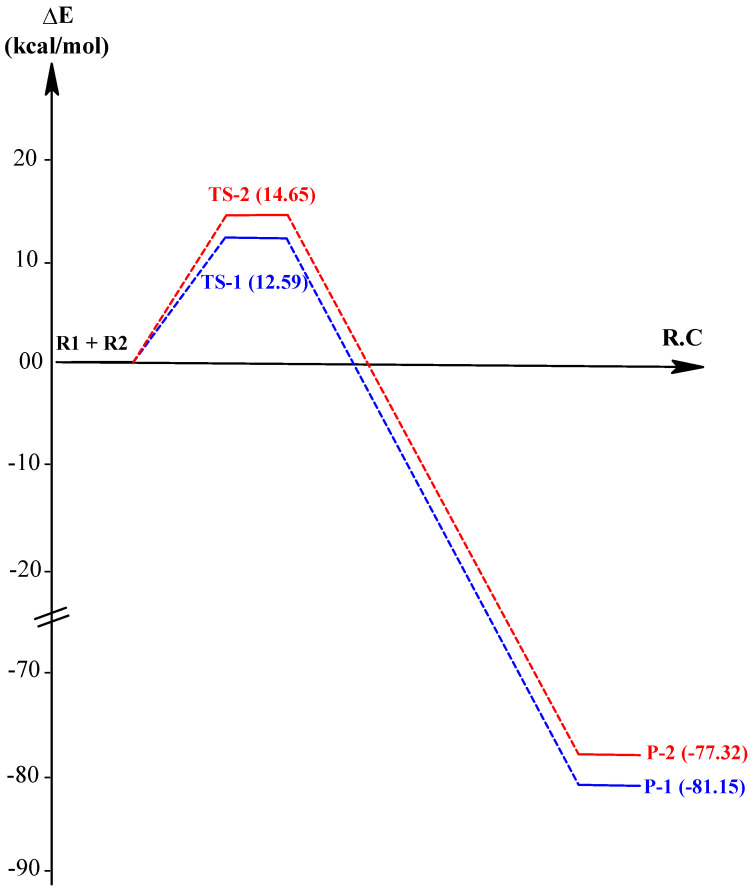
Energy profiles for the studied reaction paths of the 1,3-dipolar cycloaddition reaction between **3** and **4e**. Red indicates transition state-2, and Blue indicates transition state-1.

The energies and relative energies were calculated and summarized in Table 3, and the energy profiles of the reaction paths associated with the 1,3-dipolar cycloaddition reaction between compounds **3** and **4e** are presented in Figure 8. 

According to the results presented in Table 3 and Figure 8, the 1,3-dipolar cycloaddition reaction between **3** and **4e** exhibits an exothermic character with −81.15 and −77.32 kcal. mol^−1^ for products P-1 and P-2, respectively, indicating that the formation of these products is solely under kinetic control. The activation energies associated with TS-1 and TS-2 of this reaction are 12.59 and 14.65 kcal.mol^−1^, respectively, which shows that the formation of product P-1 is kinetically favored over P-2. Consequently, P-1 is kinetically and thermodynamically favored, which is consistent with the experimental results, obtaining compound 5 instead of 5′ (Scheme 2).

**Figure 9:**
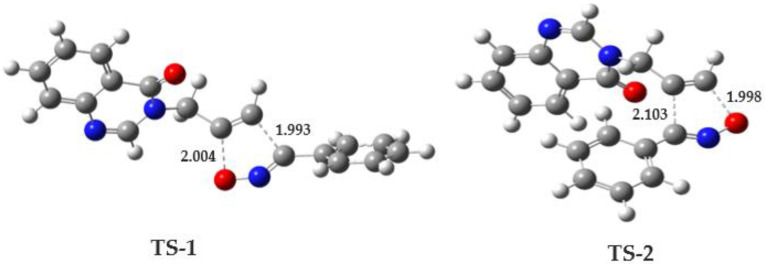
B3LYP/6-31G(d) geometries of the TSs involved in the regioisomeric pathways associated between **3** and **4e**. Distances are given in Angstroms Å. Grey indicates the carbon atom, Red indicates the Oxygen atom, and Blue indicates the Nitrogen atom.

A comparative analysis of the geometric parameters of the transition states has been conducted. For TS-1, the bond lengths for the formed bonds C_1_–O_5_ and C_2_–C_3_ are 2.004 and 1.993 Å, respectively, and for TS-2, the bond lengths for the formed bonds C_1_–C_3_ and C_2_–O_5_ were 2.103 and 1.998 Å, respectively. This indicates that the new single bonds are formed asynchronously. The geometries of the transition states associated with the two reaction pathways are presented in Figure 9.

The Intrinsic Reaction Coordinate (IRC) curve is essential for elucidating reaction mechanisms, as it illustrates the progression of the molecular structure from the initial to the final state, traversing through the transition state. Analysis of the IRC curve for our 1,3-dipolar cycloaddition reaction, specifically for the favored transition state TS-1, reveals that the reaction proceeds in a single step without the formation of intermediates, indicating an asynchronous concerted mechanism.

## 3. Materials and Methods

### 3.1. Chemistry

The new compounds **5a–e** were obtained according to the protocols described in the Supplementary Materials, which also provides detailed information on the reagents, solvents, synthesis methods and analysis techniques used.

### 3.2. Crystallographic Study

Single crystals of **5a** and **5c** were obtained at room temperature by vapor diffusion between a dichloromethane solution of the respective compound and pentane. The crystals of **5a** and **5c**, were mounted on MicroMount cryoloops (MiTeGen, Ithaca, NY, USA) and data were collected on a Bruker D8 VENTURE diffractometer (Karlsruhe, Germany) using Mo-Kα radiation (λ = 0.71073 Å) from a IμS 3.0 micro focus source with multilayer optics, at low temperature (100 K). For structure solving and refinement the Bruker APEX4 software package was used [48]. The structures were solved by dual methods (SHELXT-2018/2) [49] and refined by full matrix least-squares procedures based on F2 with all measured reflections (SHELXL-2019/1) [50]. The structures were refined with anisotropic thermal parameters for non-H atoms. Hydrogen atoms were placed in fixed, idealized positions and refined with a riding model and a mutual isotropic thermal parameter. 

Further details on the data collection and refinement methods can be found in Table 1 in the main text. The drawings were created with the Diamond program (version 5.02) [51]. The CCDC reference numbers are **2371662** (**5a**), **2371661** (**5c**). The supplementary crystallographic data for this paper can be obtained free of charge from The Cambridge Crystallographic Data Centre via https://www.ccdc.cam.ac.uk/structures/ (accessed on 25 September 2024).

### 3.3. Antioxidant Assessment

The antioxidant test was carried out using the 2.2-diphenyl-1-picrylhydrazyl radical (DPPH) method as reported in our previous study [52]. Thus, 2.5 mL of each compound at different concentrations (10–400 μg/mL) was added to 0.5 mL of a methanolic solution of DPPH (0.2 mM). Absorbance is read against a blank prepared for each concentration at 517 nm after 30 min incubation in the dark at room temperature on a UV-VIS spectrophotometer. The positive control was a solution of a standard antioxidant, ascorbic acid, whose absorbance was measured under the same conditions as the samples. For each concentration, the test was repeated 3 times. Results were expressed as percent inhibition (I%).

I% = [(Abs control − Abs tested)/Abs control] ∗ 100(1)

where Abs control is the absorbance of the negative control, and Abs tested is Absorbance of the test sample at 30 min. The tests were done in triplicate and the half maximal inhibitory concentration (IC_50_) values were reported as means ± SD.

### 3.4. Computational Methods

DFT calculations were performed using the B3LYP functional [53] combined with the 6-31G(d) basis set [54]. Optimization was achieved through the Berny analytical gradient method [55]. Stationary points were verified by frequency calculations to ensure that transition states (TSs) exhibited precisely one imaginary frequency. Intrinsic Reaction Coordinate (IRC) paths [56] were mapped to illustrate the energy profiles connecting each TS to the corresponding minima [57]. All computations were carried out with the Gaussian 09 suite of programs [58]. The global electrophilicity index ω [59] was calculated using the formula ω = (μ^2^/2η), where μ represents the electronic chemical potential and η denotes the chemical hardness. These quantities are derived from the one-electron energies of the frontier molecular orbitals, HOMO and LUMO, with μ defined as (EHOMO − ELUMO)/2 and η (ELUMO − EHOMO), respectively [60]. We also introduced an empirical nucleophilicity index (N) [61], based on HOMO energies within the Kohn-Sham framework [62], defined as N = EHOMO(Nu) − EHOMO(TCE). The nucleophilicity is referenced to tetracyanoethylene (TCE), which has the lowest HOMO energy among a wide range of organic molecules considered [61]. This reference allows for a convenient positive nucleophilicity scale.

## 4. Conclusions

In conclusion, we have successfully synthesized a new series of quinazolin-4(3H)-one-isoxazole molecules **5a–e**, confirming their structures by characterization techniques such as infrared spectroscopy (IR), proton and carbon-13 nuclear magnetic resonance, 2D HMBC NMR, mass spectrometry, and corroborated by crystallographic studies. Preliminary studies on antioxidant activity show that the newly synthesized compounds do not exhibit significant efficacy as antioxidant agents. These results indicate that, although the basic structure is promising, further modifications are required to enhance their biological activity. The regioselectivity and mechanism of the 1,3-dipolar cycloaddition reaction between compound **3** and compound **4e** were studied using DFT calculation methods at the B3LYP/6-31G(d) level. The study of the energy profiles associated with this reaction demonstrated that the formation of the two products P-1 and P-2 is exothermic by −81.15 and −77.32 kcal.mol^−1^, respectively, and indicating that P-1 is kinetically and thermodynamically favorable, in good agreement with the experimental results. Additionally, the IRC curve showed that the reaction follows an asynchronous concerted mechanism.

## Figures and Tables

**Table 1:** Crystal data and refinement details for compounds **5a** and **5c**.

Compound	5a	5c
Molecular formula	C_18_H_12_BrN_3_O_2_	C_19_H_15_N_3_O_2_
Formula weight	382.22	317.34
Crystal size/mm^3^	0.145 × 0.095 × 0.055	0.216 × 0.186 × 0.175
Crystal habit	colorless block	colorless block
*λ*(Mo*K*_α_)/Å	0.71073	0.71073
*T*/K	100.(2)	100.(2)
Crystal system	monoclinic	orthorhombic
Space group	*Pc*	*Pbca*
*a*/Å	4.5517(5)	11.4436(3)
*b*/Å	16.0529(16)	7.8897(3)
*c*/Å	10.6519(10)	33.7363(12)
*α*/°	90	90
*β*/°	100.283(4)	90
*γ*/°	90	90
*V*/Å^3^	765.81(13)	3045.93(18)
*Z*	2	8
*D*_calc_/g cm^−3^	1.658	1.384
*μ*/mm^−1^	2.700	0.092
*θ* range for data collections (°)	2.32–28.28	2.15–28.28
*F*(000)	384	1328
*T*_max_/*T*_min_	0.746/0.629	0.746/0.694
Refl. collected/unique/*R*_int_	60212/3719/0.0273	37141/3783/0.0287
Completeness to *θ*	99.9%	99.9%
Refinement method	Full-matrix least-squares on F^2^	
Data/restraints/parameters	3719/2/221	3783/0/221
Goodness-of-fit, *S*	1.092	1.068
Final *R* indices [*I* > 2*σ*(*I*)]	*R*_1_ = 0.0133	*R*_1_ = 0.0291
	*wR*_2_ = 0.0312	*wR*_2_ = 0.0792
*R* indices (all data)	*R*_1_ = 0.0141	*R*_1_ = 0.0321
	*wR*_2_ = 0.0316	*wR*_2_ = 0.0820
Δ*ρ*_max_, Δ*ρ*_min_/*e* Å^−3^	0.231; −0.121	0.299; –0.169
CCDC No.	2371662	2371661

**Table 2:** B3LYP/6-31G(d) electronic chemical potential µ, chemical hardness η, electrophilicity ω, nucleophilicity N, in eV, of the two reagents.

System	µ	η	ω	N
**3**	−3.76	2.56	2.77	2.80
**4e**	−3.83	2.51	2.91	2.78

**Table 3:** B3LYP/6-31G(d) total energy (E, in a.u.) and relative energy (ΔE, in kcal/mol), for the species involved in the 1,3-dipolar cycloaddition reaction between **3** and **4e**.

	E (u.a)	ΔE (kcal/mol)
	−608.664	-------
R1 (3)	−399.640	-------
R2 (4e)	−1008.304	-------
R1 + R2	−1008.433	–81.15
P-1	−1008.427	–77.32
P-2	–1008.284	12.59
TS-1	–1008.280	14.65

## Data Availability

The original contributions presented in the study are included in the article/Supplementary Materials, further inquiries can be directed to the corresponding authors.

## Supplementary Materials

The following supporting information can be downloaded at: https://www.mdpi.com/article/10.3390/ph17101390/s1. Reference [39] is cited in the Supplementary Materials.
