# Supplementary material for: New Quinazolin-4(3H)-One Derivatives Incorporating Isoxazole Moiety as Antioxidant Agents: Synthesis, Structural Characterization, and Theoretical DFT Mechanistic Study
Source: Pharmaceuticals (Basel). 2024 Oct 18;17(10):1390. doi: 10.3390/ph17101390 (PMC11510333; doi:10.3390/ph17101390)
Supplement: Supplementary file 1 [file pharmaceuticals-17-01390-s001.zip › pharmaceuticals-3251571-supplementary.pdf]

1. Materials and methods

1.1.Chemical reagents and instruments

Commercial reagents were used without further purification, and reactions were monitored by thin-layer chromatography (TLC) to determine the outcome. Column chromatography was performed using Merck silica gel (100-200 mesh). A thin-layer chromatography plate (silica gel 60 F254) was used to determine the purity of the compound, and UV light was used to visualize the compound (VILBER LOURMAT, VL-215.LC). A KOFLER bench was used to determine the melting points of the materials with an accuracy of ± 2°C. A Bruker Avance II spectrometer was used to acquire proton NMR spectra at 300 MHz, and carbon NMR spectra at 75 Hz. TMS was used as a reference for NMR measurements. Chemical shifts are expressed in parts per million (ppm). The NMR assignment for product **5c** was confirmed by HMBC experiments (1H-13C spectroscopic correlation). Mass spectrometry (MS) spectra were obtained using a Hewlett-Packard 5989A spectrometer in electron impact mode (70 eV). HRMS were performed on Thermo Scientific LTQ Orbitrap XL mass spectrometer ESI or APCI ionization mode. FT-IR spectra were recorded on a Bio-rad FTS-135 spectrophotometer using KBr pellets, with absorption maxima indicated in cm^{-1}.

1.2.Synthetic procedures

quinazolin-4(3H)-one (2): The quinazolin-4(3H)-one (2) was prepared using the following previously reported procedure[1]. In a 100 mL flask equipped with a condenser anthranilic acid (1,37 g, 10 mmol) and formamide (16,8 mmol, 0,67 mL) was refluxed at 130-140°C for 4h. Then, the formed precipitate was isolated by filtration and recrystallized in ethanol to obtain 1,31 g of quinazolin-4(3H)-one 2 as white solid. Yield: 92%, p.m.: 214-216°C. ^{1}H NMR (300 MHz, DMSO-d_6 δ in ppm): 12.27 (s, 1H), 8.13 (ddd, *J* = 8.0, 1.6, 0.6 Hz, 1H), 8.11 (s, 1H), 7.88 – 7.76 (m, 1H), 7.67 (ddd, *J* = 8.2, 1.3, 0.6 Hz, 1H), 7.53 (ddd, *J* = 8.2, 7.1, 1.2 Hz, 1H). 13C NMR (75 MHz, DMSO-d6, δ in ppm):

161.23, 149.16, 145.90, 134.79, 127.64, 127.22, 126.30, 123.08. ESIMS (m/z): found 146,05, calculated 147,0555 for $[M-H]^+$.

3-(prop-2-yn-1-yl)quinazolin-4(3H)-one (3) : In 100 mL round-bouttom flask, quinazolin-4(3H)-one **(2)** (10 mmol, 1.47 g), K_2CO_3 , and TBAB (1.2 equivalents) were dissolved in 15 ml of DMF. The reaction mixture was stirred for 30 minutes at room temperature, then propargyl bromide (1.2 equivalents) was added to the mixture and the stirring was continued until the consumption of the reagents. Once the reaction was complete, the resulting mixture was diluted with 100 ml of cold water. The formed precipitate was isolated by filtration, dried, and purified by column chromatography with a mixture of ethyl acetate /hexane (1/3) as eluent to obtain 3 as white solid (1.35 g, yield: 73 %), m.p: 116-118 °C. ^{1}H NMR (300 MHz, $CDCl_3$, δ inppm): 8.37-8.33 (m, 2H), 7.84-7.75 (m, 1H), 7.58-7.52 (m, 1H), 4.85 (d, 2H, J = 2.7 Hz), 2.52 (t, 1H, J = 2.7Hz). ^{13}C NMR: δ(75 MHz, $CDCl_3$, d in ppm): 160.31, 147.55, 145.12, 134.65, 127.69, 127.41, 126.88, 121.71, 75.31, 35.30.

General procedure for the synthesis of the quinazoline-4(3H)-one –isoxazole derivatives 5a–e:

In a 100 ml flask, dipolarophile **(3)** (1 mmol, 1.84g) and arylhydroxamoyl chloride **4a-e** (1.2 mmol, g) of were dissolved in 40 ml of chloroform. Subsequently, anhydrous triethylamine (1.2 mmol, mL) was added dropwise. After the addition was complete, the reaction mixture was maintained at room temperature with magnetic stirring for the appropriate duration. Once the reaction was complete, as indicated by TLC, the reaction mixture was transferred to a separatory funnel and washed three times with 20 mL of water. The organic layer was dried over anhydrous sodium sulfate (Na_2SO_4), filtered, and the solvent was then removed by rotary evaporation. The resulting residue was purified on a silica gel column using a mixture of hexane and ethyl acetate (3:1) as eluent.

3-((3-(4-bromophenyl)isoxazol-5-yl)methyl)quinazolin-4(3H)-one (5a) : Yield (85%); m.p.: 135 °C, IR (u in cm^{-1}): 3018 (=C–H), 2358 (C–H), 1672 (C=O), 1612 (C=N), 1473,

1425, 1471 (C=C), 1214 (C–O); ^{1}H NMR (300 MHz, DMSO δ in ppm): 8.59 (s, 1H, N=CH–N), 8.19-8.17 (d, 1H, ^{3}J= 7.8 Hz , Ar–H), 7.91-7.57 (m, 7H, Ar–H), 7.1 (s, 1H, C<u>H</u>$_{isoxazole}$), 5.45 (s, 2H, N–C<u>H</u>2). ^{13}C NMR (75 MHz, DMSO δ in ppm): 169.12 (C–O$_{isoxazole}$), 161.76 (C=N$_{isoxazole}$), 160.4 (C=O$_{amide}$), 148.34, 148.08, 135.18, 132.58, 129.11, 127.93, 127.88, 127.83, 126.64, 124.25, 122.01, 101.56, 42.04 (<u>C</u>H$_2$). ESI-QTOF-MS (*m*/*z*): mass calculated for $[C_{18}H_{13}N_3O_2Br]^+$ 382.0191, mass found 382.0182.

3-((3-(4-chlorophenyl)isoxazol-5-yl)methyl)quinazolin-4(3H)-one (5b): Yield:72%, m.p: 178 °C, IR (u in cm^{-1}): 3118 (=C-H), 1670 (C=O), 1598 (C=N), 1425, 1361 (C=C), 1150 (C–N), ^{1}H NMR (300 MHz, DMSO, δ in ppm): 8.59 (s, 1H, N=CH–N), 8.19-8.16 (dd, 1H, Ar–H, ^3J_o = 8 Hz, ^4J_m =1.5 Hz,), 7.91–7.85 (m, 3H, Ar–H), 7.75–7.73 (d, 1H, Ar–H, J= 6 Hz), 7.62–7.54 (m, 3H, Ar–H), 7.1 (s, 1H, CH$_{isoxazole}$), 5.45 (s, 2H, N–CH2). ^{13}C NMR (75 MHz, DMSO, in δ ppm): 169.1 (C–O$_{isoxazole}$), 161.67 (C=N$_{isoxazole}$), 160.40 (C=O$_{amide}$), 148.07, 135.49, 135.17, 129.66, 128.90, 127.87, 127.58, 126.63, 122.01, 101.58, 42.04 (<u>C</u>H$_2$). HRMS (*m*/*z*): mass calculated for $[C_{18}H_{13}N_3O_2Cl]^+$ 338.0696, mass found 338.0688.

3-((3-(p-tolyl)isoxazol-5-yl)methyl)quinazolin-4(3H)-one (5c) : Yield (65%); m.p.: 162 °C, IR (u in cm^{-1}): 3114 (=C-H), 2918 (C–H), 1679 (C=O), 1600 (C=N), 1562, 1473, 1422 (C=C), 1159 (C–O); ^{1}H NMR (300 MHz, $CDCl_3$ δ in ppm): 8.35-8.32 (m, 1H, Ar–H), 8.25 (s, 1H, N=C<u>H</u>–N), 7.84-7.74 (m, 2H, Ar–H), 7.55 (tdd, 1H, ^{3}J= 7.5 , ^{3}J= 7.35 , ^{4}J=1.35 Hz ; Ar–H), 6.67 (s, 1H, C<u>H</u>$_{isoxazole}$), 5.35 (s, 2H, N–C<u>H</u>2) , 2.4 (s, 3H, C<u>H</u>$_3$). ^{13}C NMR (75 MHz, $CDCl_3$ δ in ppm): 166.02 (C–O$_{isoxazole}$), 162.87 (C=N$_{isoxazole}$), 160.65 (C=O$_{amide}$), 148.03, 145.61, 140.47, 134.72, 129.64, 127.81, 127.73, 126.82, 126.74, 125.58, 121.96, 102.2, 41.18 (<u>C</u>H$_2$), 21.42 (<u>C</u>H$_3$). HRMS (*m*/*z*): mass calculated for $[C_{19}H_{16}N_3O_2]^+$ 318.1242, mass found 318.1236.

3-((3-phenylisoxazol-5-yl)methyl)quinazolin-4(3H)-one (5d) : Yield (55%); m.p : 144 °C, IR (u in cm^{-1}): 3018 (=C–H), 2359 (C–H), 1684 (C=O), 1613 (C=N), 1496, 1457 (C=C), 1159 (C–O), ^{1}H NMR (300 MHz, $CDCl_3$ δ in ppm): 8.34 (dd, 1H, ^{3}J= 8.1 , ^{4}J= 0.9 Hz , Ar–H), 8.26 (s, 1H, N=CH–N), 7.82-7.75 (m, 4H, Ar–H), 7.56 (tdd, 1H, ^{3}J= 7.5 , ^{3}J= 7.35 Hz ,

^4J= 1.35 Hz , Ar–H), 7.47 -7.44 (m, 3H, Ar–H)), 6.7 (s, 1H, C$\underline{H}_{isoxazole}$), 5.36 (s, 2H, N–C$\underline{H}$2) , 2.4 (s, 3H, C$\underline{H}_3$). ^{13}C NMR (75 MHz, CDCl3 δ in ppm): 166.14 (C–$O_{isoxazole}$), 145.60, 134.76, 130.28, 128.95, 127.81, 127.77, 126.86, 126.82, 102.3, 41.21 ($\underline{C}H_2$). HRMS (*m*/*z*): mass calculated for $[C_{18}H_{14}N_3O_2]^+$ 304.1086, mass found 304.1072.

3-((3-(2-chlorophenyl)isoxazol-5-yl)methyl)quinazolin-4(3H)-one (5e): Yield (78%); m.p.: 135 °C, IR (u in cm^{-1}): 3100 (=C-H), 1676 (C=O), 1603 (C=N), 1556, 1500, 1470 (C=C), 1470 (C–O), 1260 (O–N), 1154 (C–N), ^{1}H NMR (300 MHz, $CDCl_3$ δ in ppm): 8.33 (t, 1H, N=C$\underline{H}$–N, ^4J = 1.2 Hz), 7.83-7.81 (m, 2H, Ar–H), 7.71-7.68 (m, 1H, Ar–H), 7.6–7.55 (m, 1H, Ar–H), 7.5–7.47 (m, 1H, Ar–H), 7.43–7.32 (m, 2H, Ar–H), 6.89 (s, 1H, C$\underline{H}_{isoxazole}$), 5.44 (s, 2H, N–C$\underline{H}$2). ^{13}C NMR (75 MHz, CDCl3 δ in ppm): 165.25 (C–$O_{isoxazole}$), 161.53 (C=$N_{isoxazole}$), 160.32 (C=O_{amide}), 146.99; 145.99, 134.93, 132.96, 131.11, 130.99, 130.44, 127.98, 127.72, 127.19, 127.13, 126.98, 121.77, 105.59, 41.42 ($\underline{C}H_2$), HRMS (*m*/*z*): mass calculated for $[C_{18}H_{13}N_3O_2Cl]^+$ 338.0696, mass found 338.0684.

2. ^{1}H, ^{13}C NMR and HRMS spectra of synthesized compounds

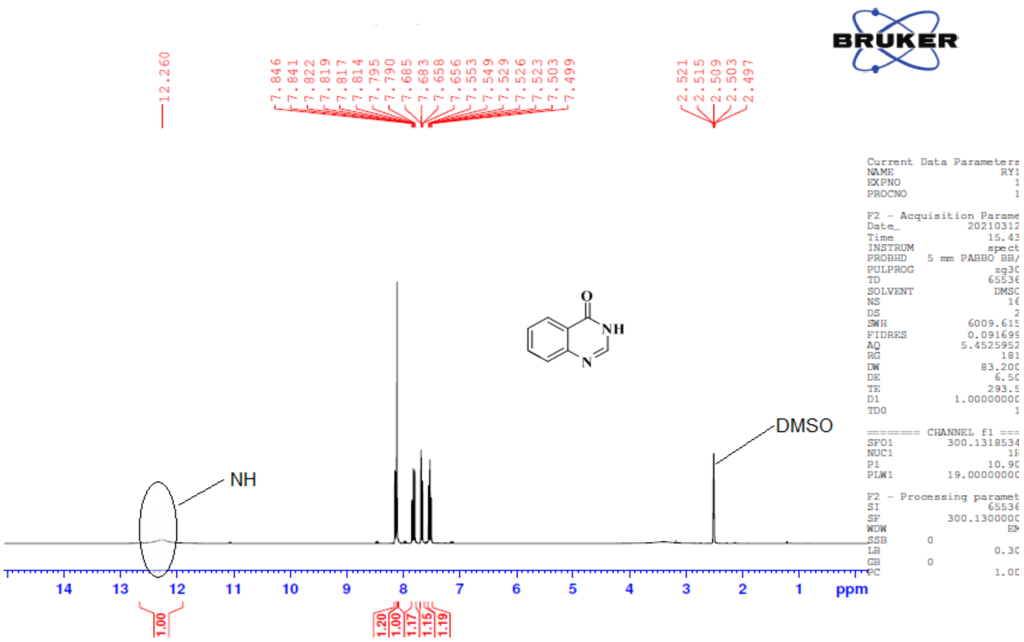

Figure S1.^{1}H NMR spectrum (300 MHz, DMSO) of compound (**2**)

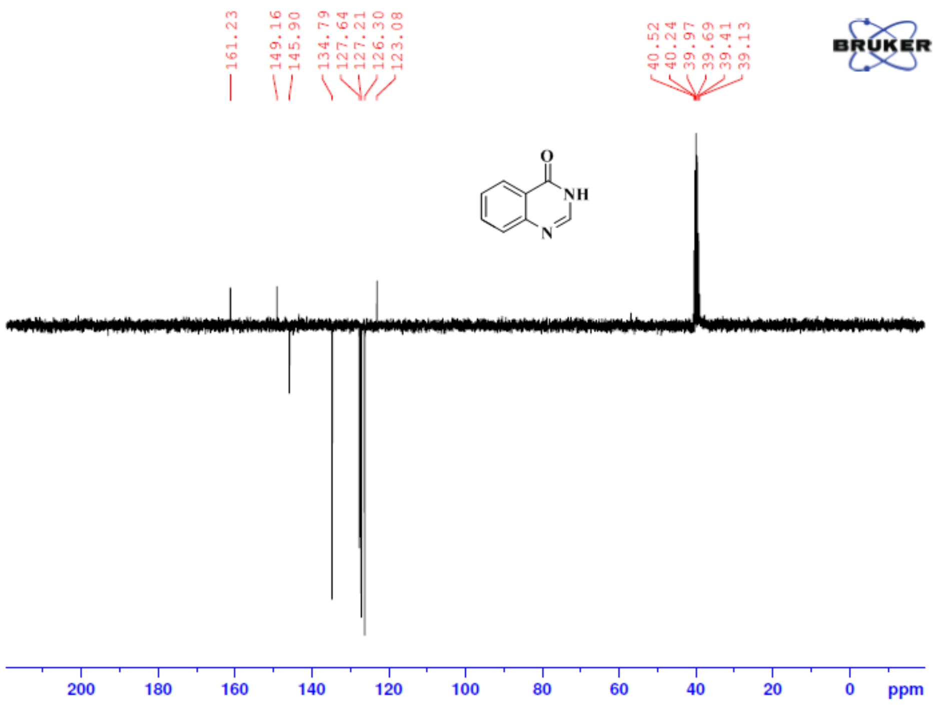

Figure S2. ^{13}C NMR spectrum (75 MHz, DMSO) of compound (**2**)

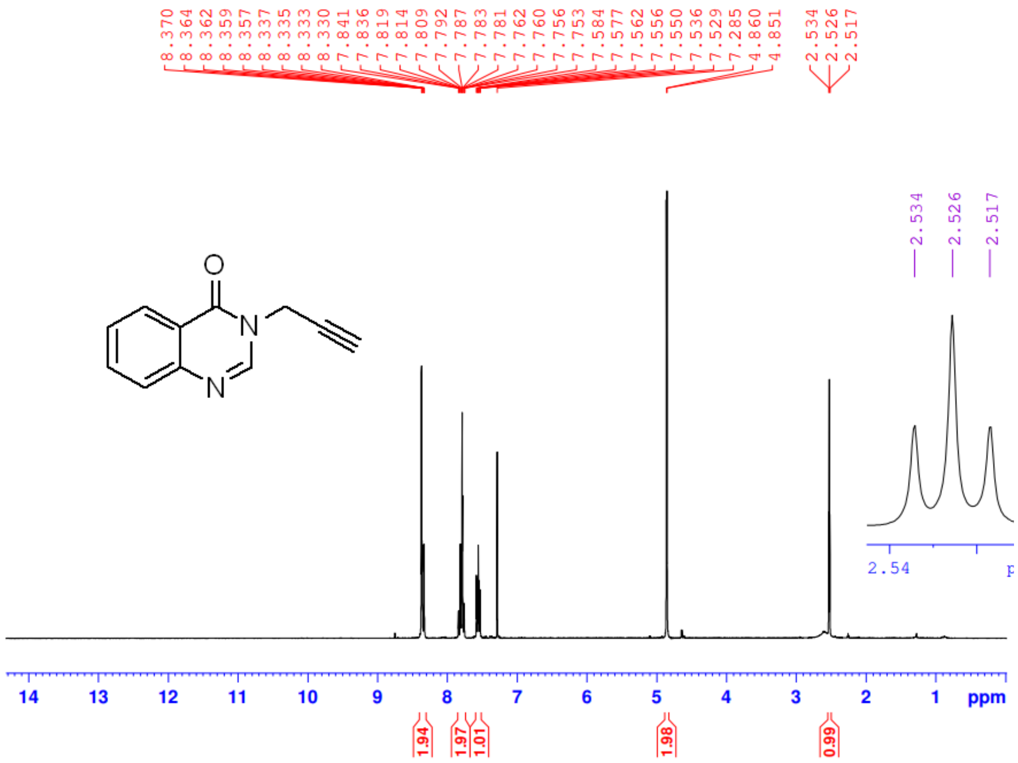

Figure S3. ^{1}H NMR spectrum (300 MHz, $CDCl_3$) of compound (**3**)

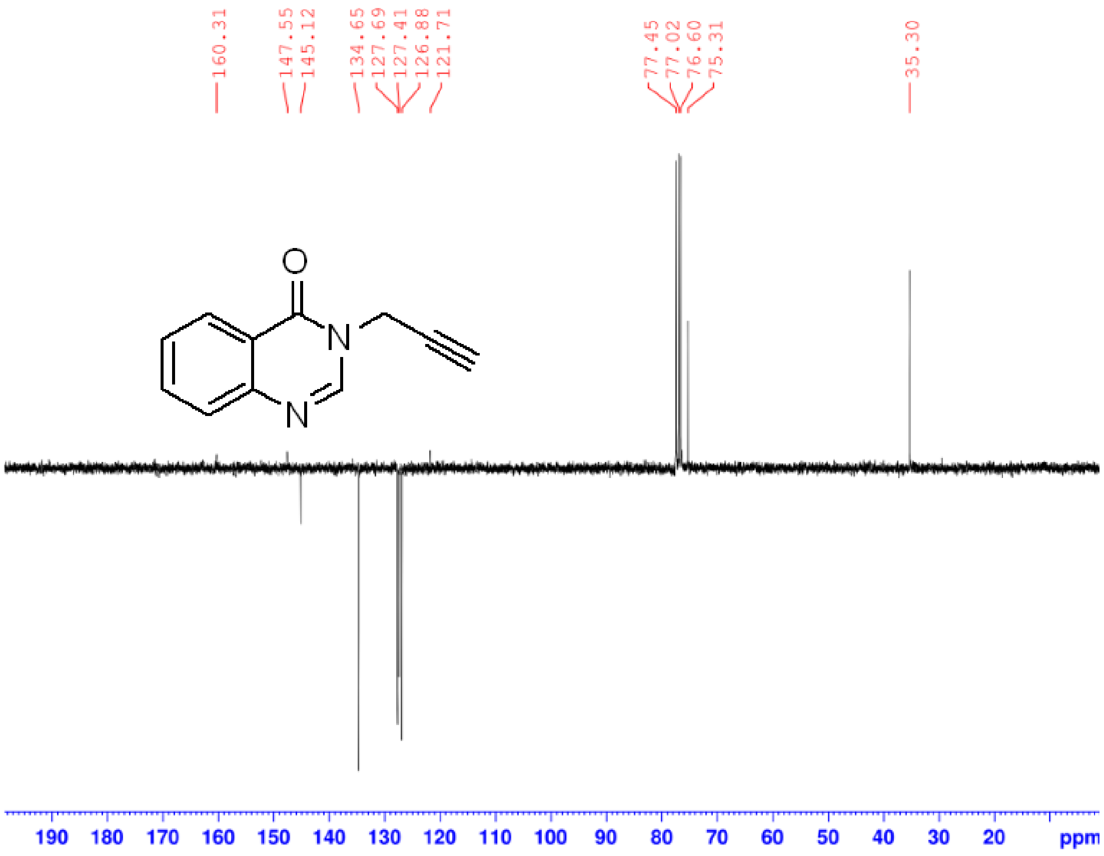

Figure S4. ^{13}C NMR spectrum (75 MHz, $CDCl_3$) of compound (**3**)

i. Hybrid compounds containing the quinazolinone and isoxazole nucleus (5a-e)

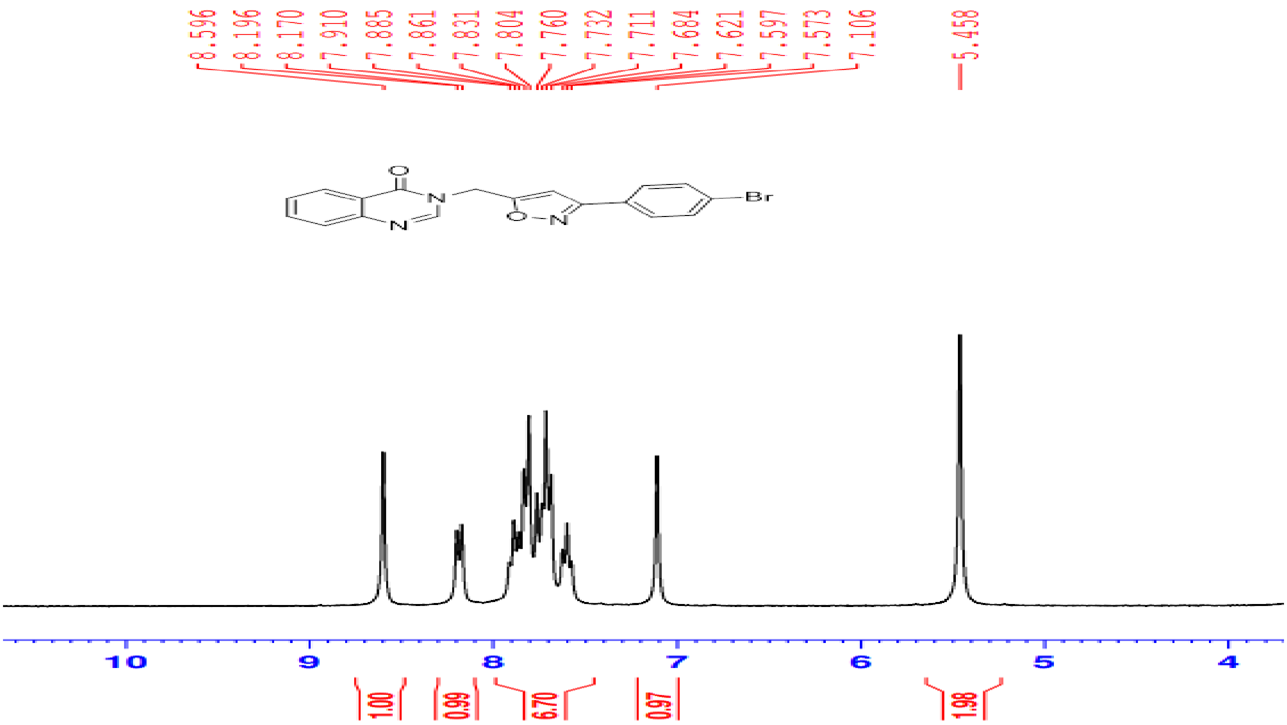

Figure S5. ^{1}H *NMR spectrum (300 MHz, DMSO) of compound* ***(5a)***

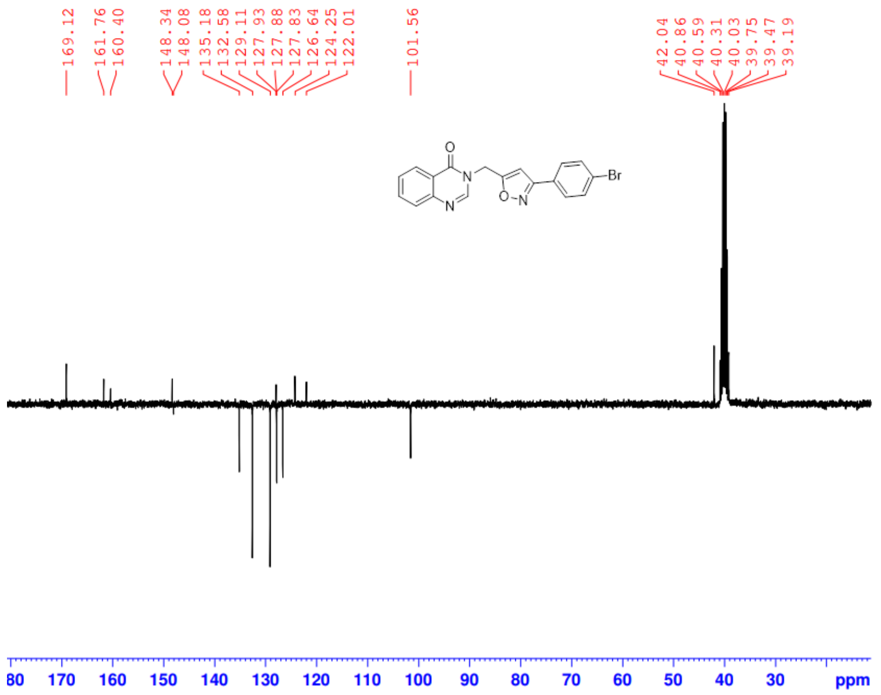

Figure S6. ^{13}C NMR spectrum (75 MHz, DMSO) of compound (**5a**)

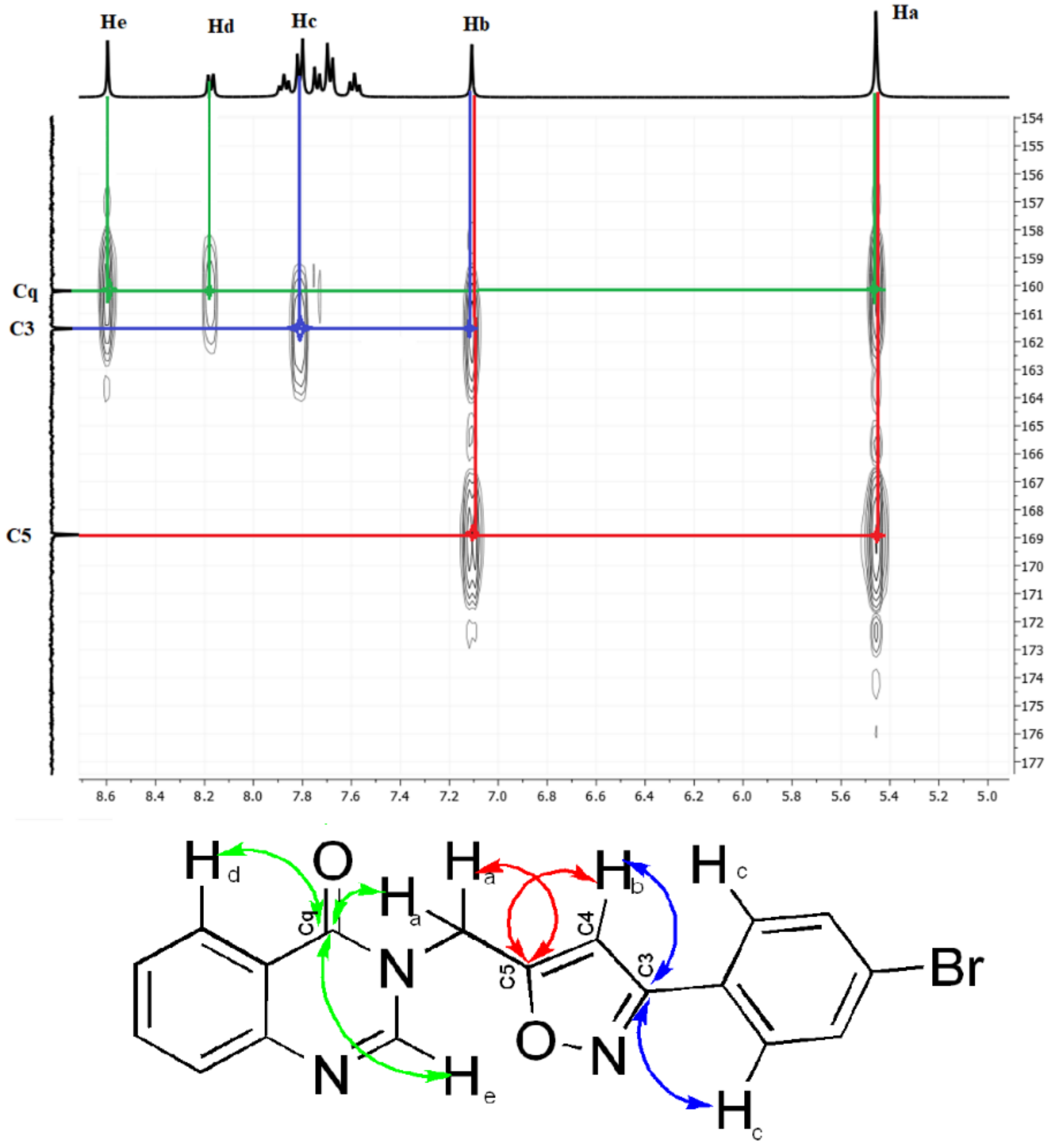

Figure S7. 2D-HMBC NMR spectrum of compound (**5a)** showing the coupling signals for hydrogen atoms.

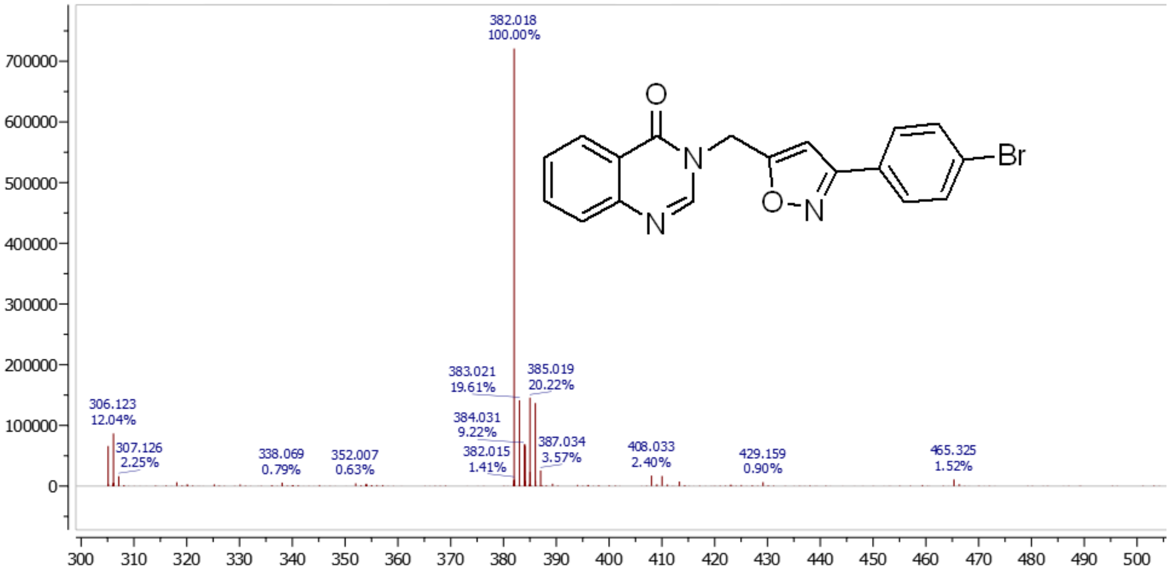

Figure S8. Mass spectrum of compound (**5a**)

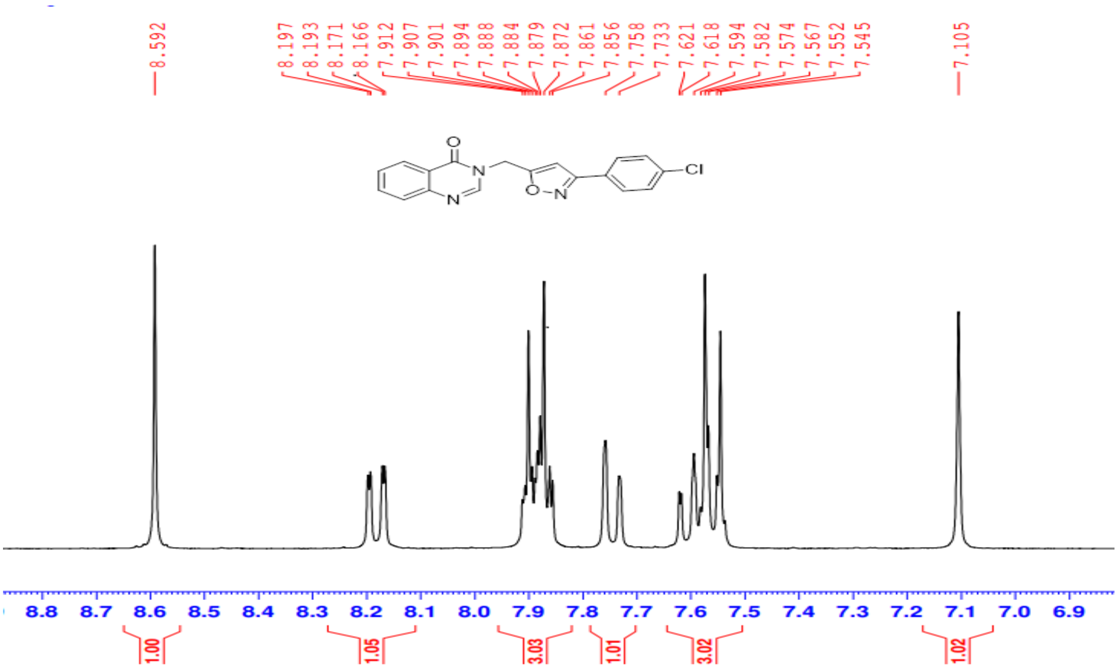

Figure 9. ^{1}H NMR spectrum (300 MHz, DMSO) of compound (**5b**)

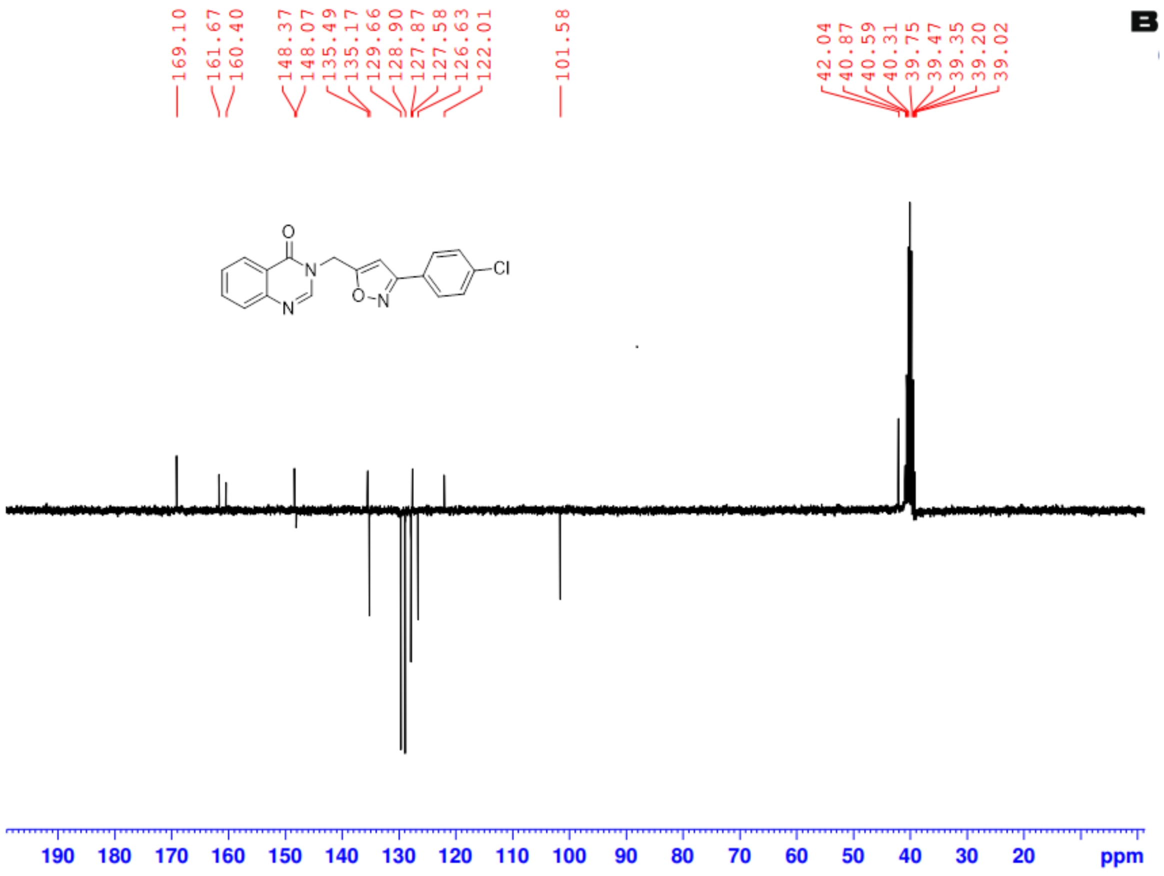

Figure 10.^{13}C NMR spectrum (75 MHz, DMSO) of compound (**5b**)

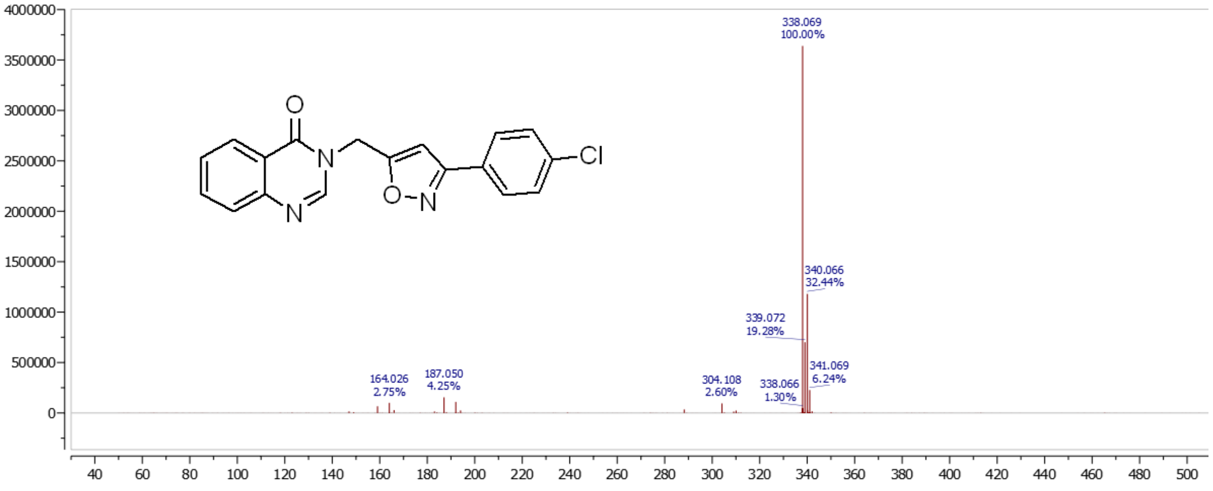

Figure S11. Mass spectrum of compound (**5b**)

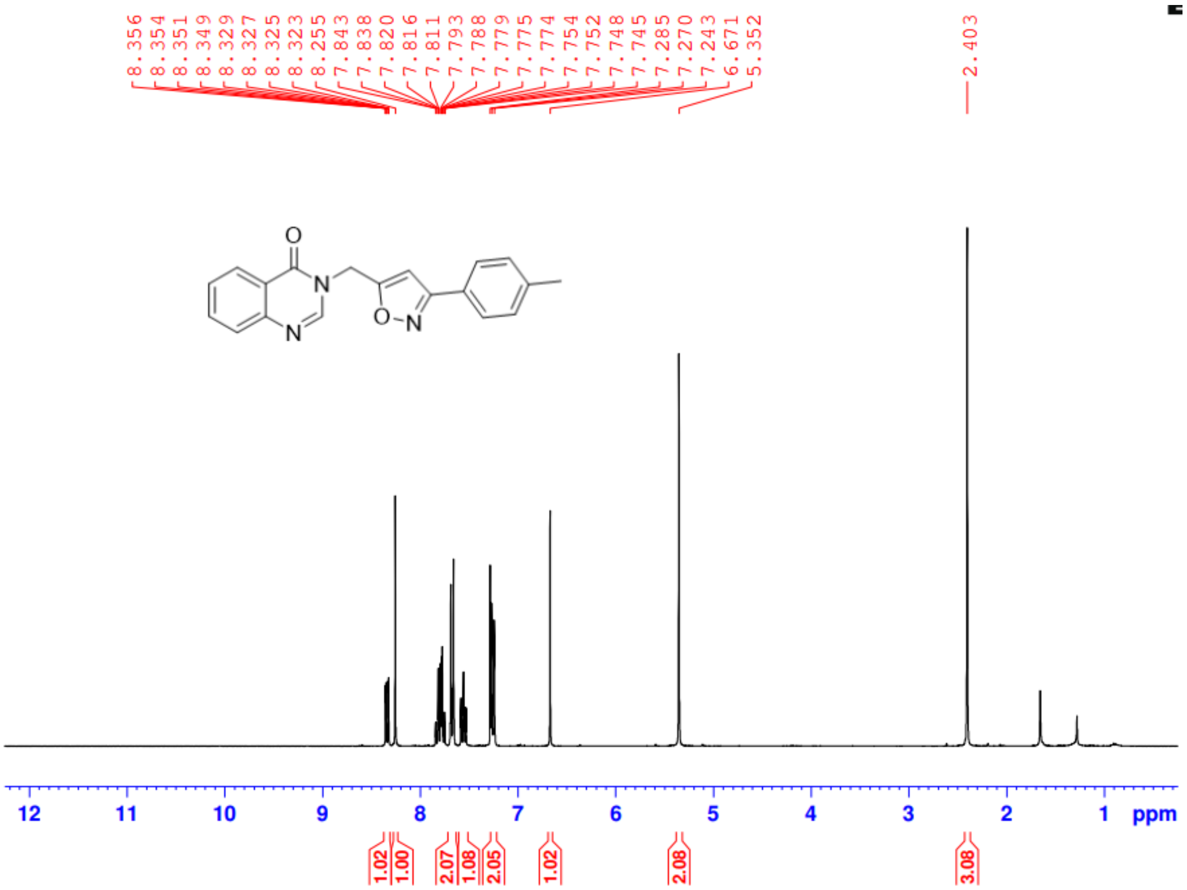
8.356
8.354
8.351
8.349
8.329
8.327
8.325
8.323
8.255
7.843
7.838
7.820
7.816
7.811
7.793
7.788
7.779
7.775
7.774
7.754
7.752
7.748
7.745
7.285
7.270
7.243
6.671
5.352
2.403
O
N
N
O
N
12
11
10
9
8
7
6
5
4
3
2
1
ppm
1.02
1.00
2.07
1.08
2.05
1.02
2.08
3.08

Figure S12. ^{1}H NMR spectrum (300 MHz, $CDCl_3$) of compound **(5c)**

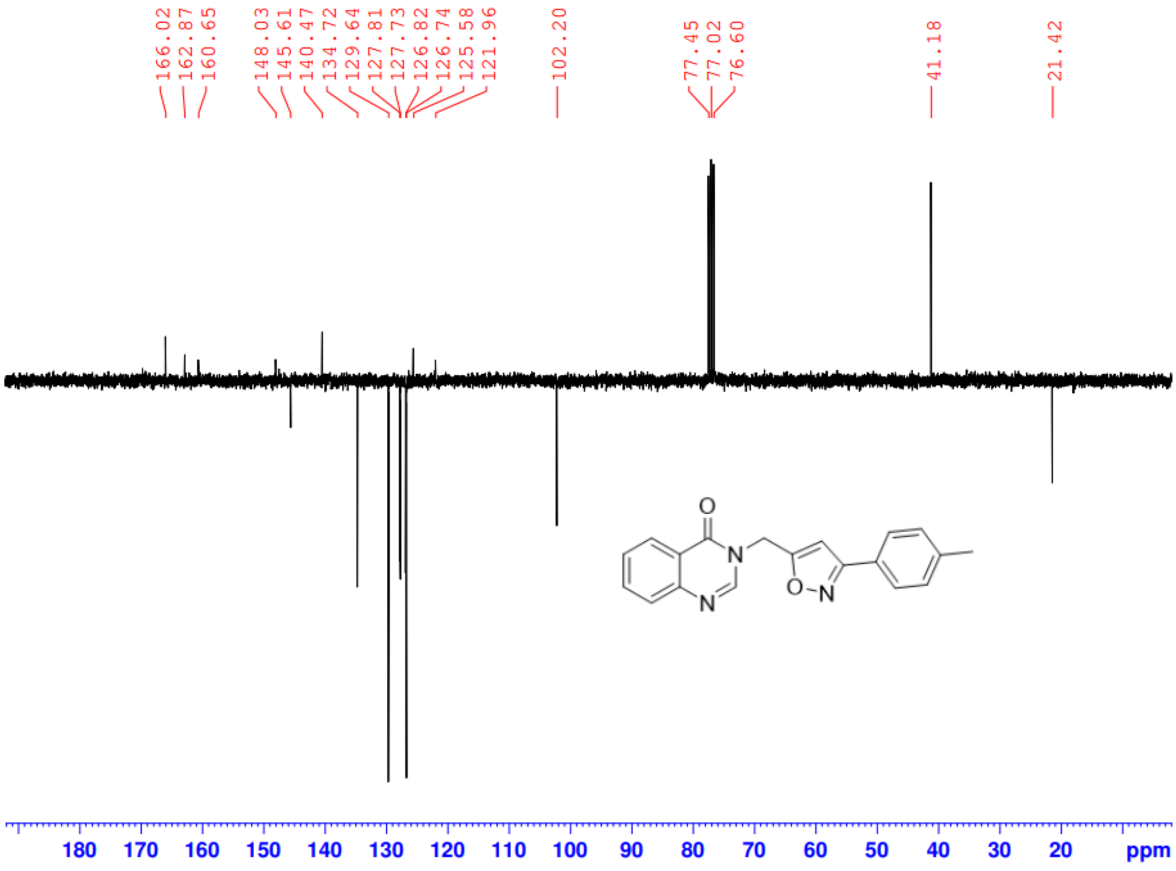

Figure S13. ^{13}C NMR spectrum (75 MHz, $CDCl_3$) of compound (**5c**)

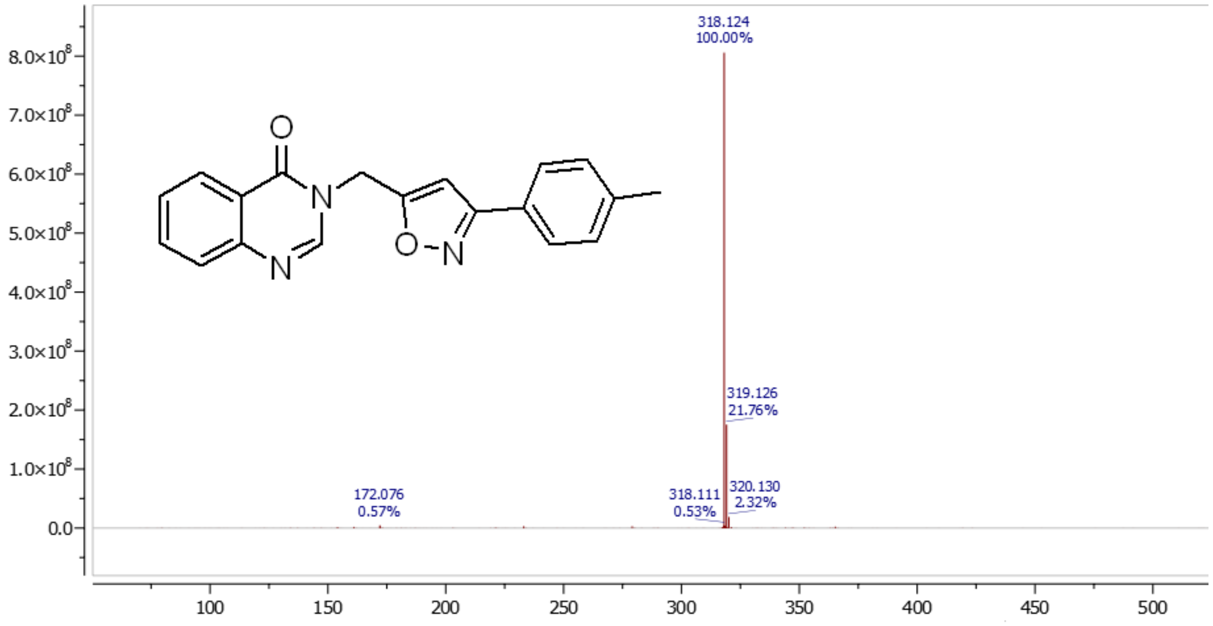

Figure S14. Mass spectrum of compound (**5c**)

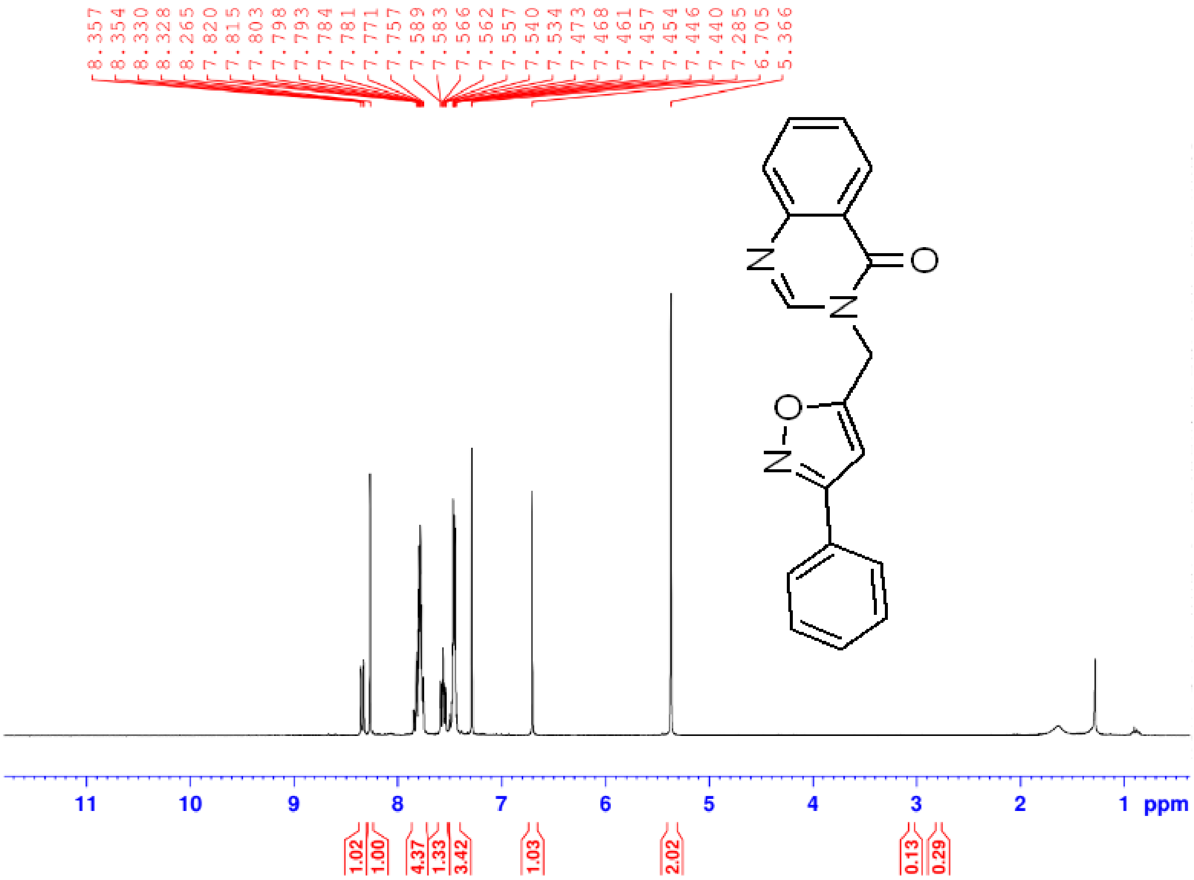

Figure S15. ^{1}H NMR spectrum (300 MHz, $CDCl_3$) of compound **(5d)**

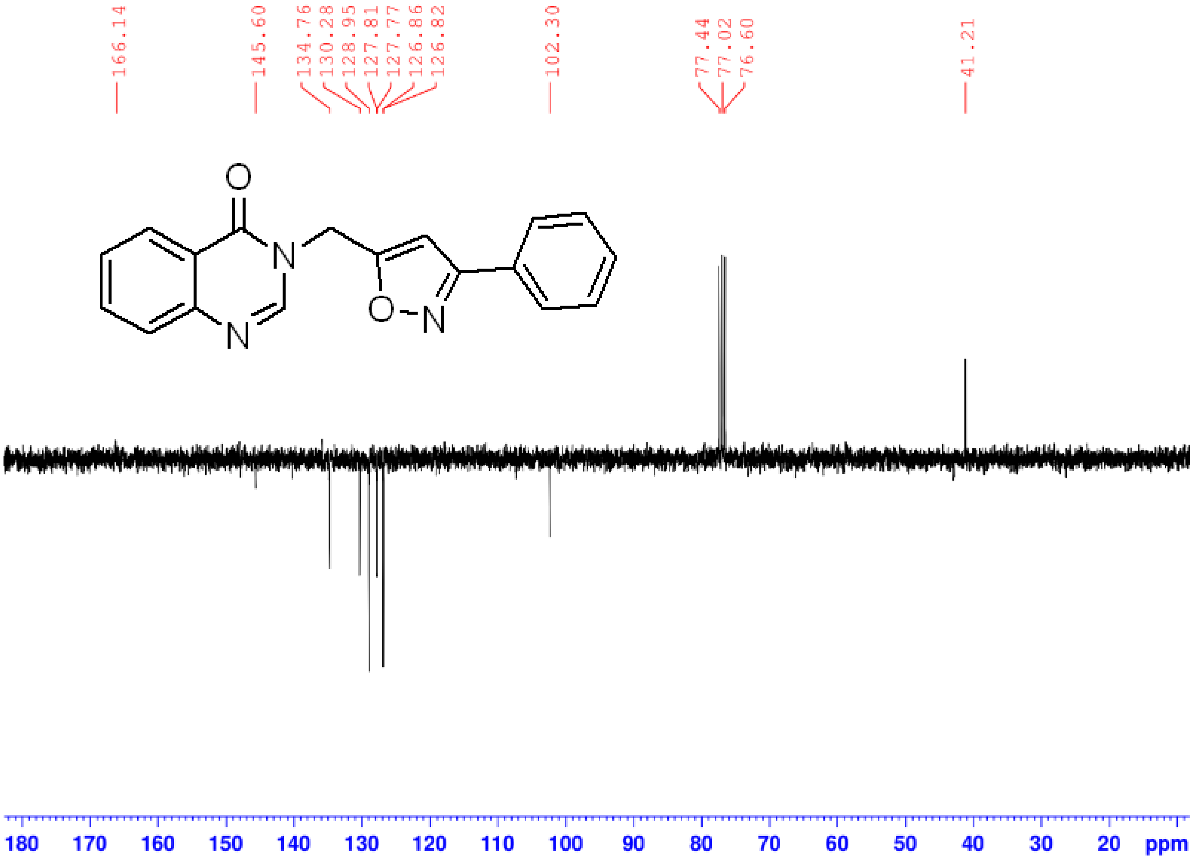

Figure S16. ^{13}C NMR spectrum (75 MHz, $CDCl_3$) of compound **(5d)**

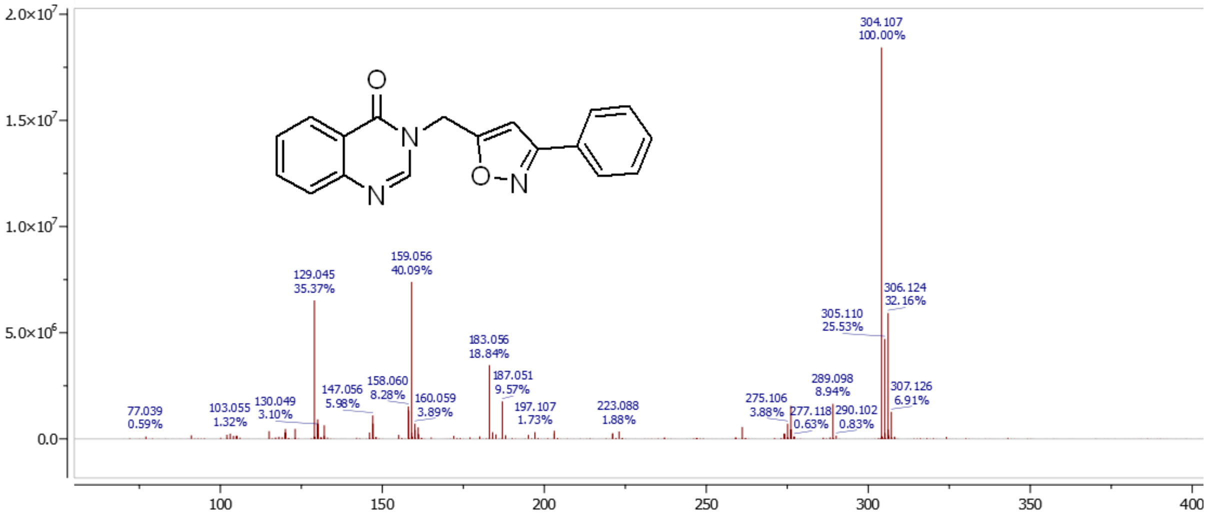

Figure S17. Mass spectrum of compound (**5d**)

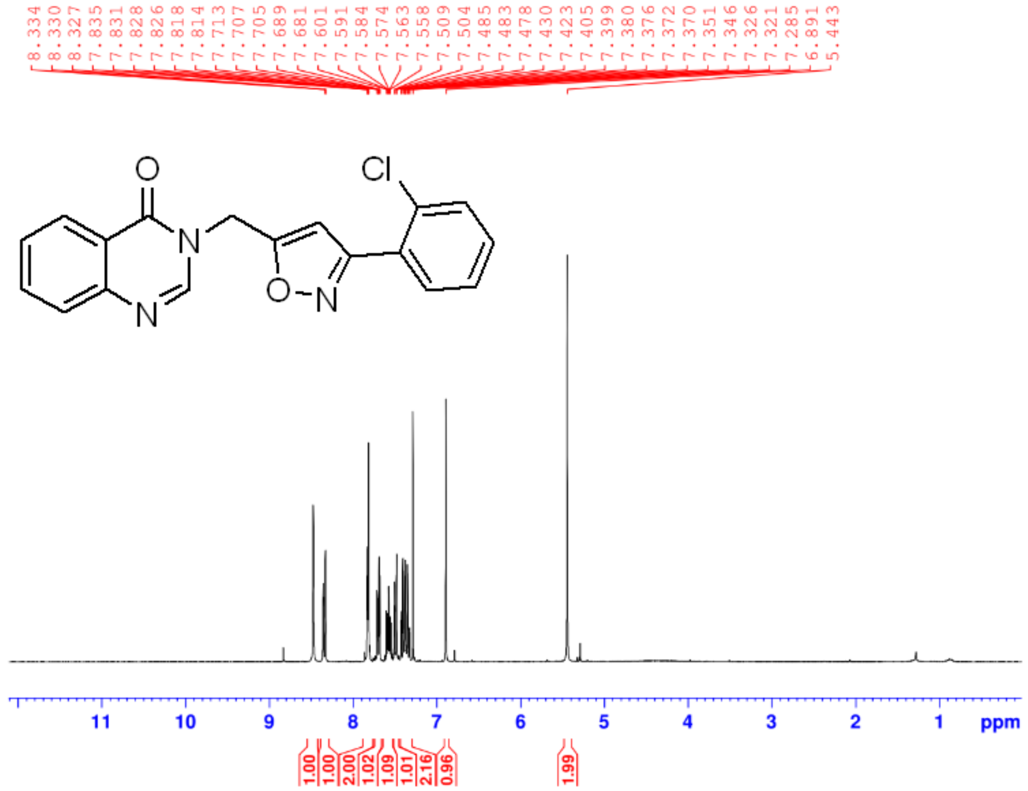

Figure S18. ^{1}H NMR spectrum (300 MHz, $CDCl_3$) of compound (**5e**)

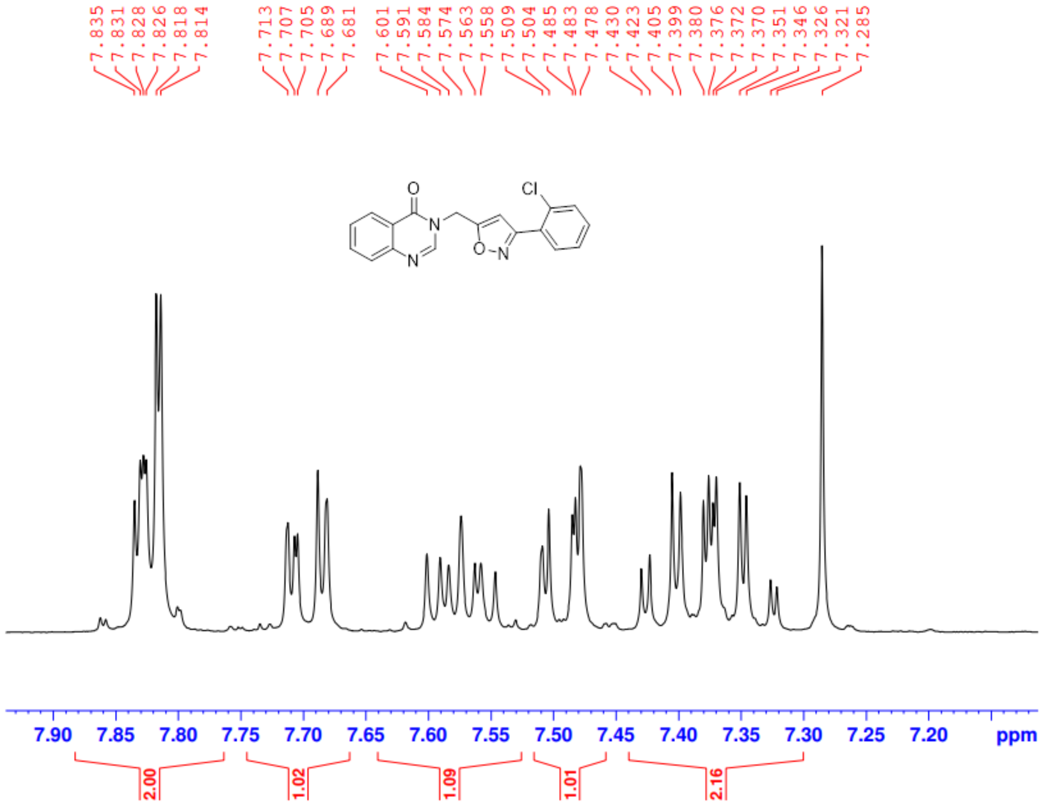

Figure S19. ^{1}H NMR spectrum (300 MHz, $CDCl_3$) of compound (**5e**)

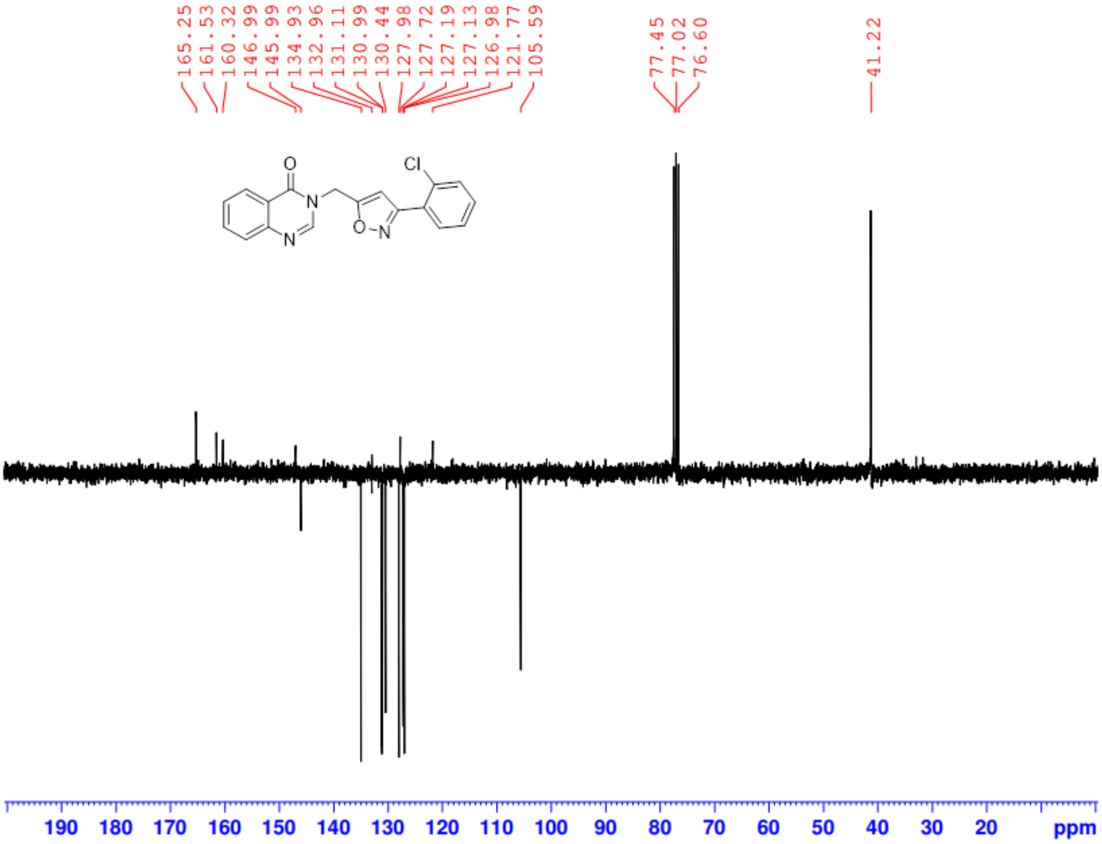

Figure S20.^{13}C NMR spectrum (75 MHz, $CDCl_3$) of compound (**5e**)

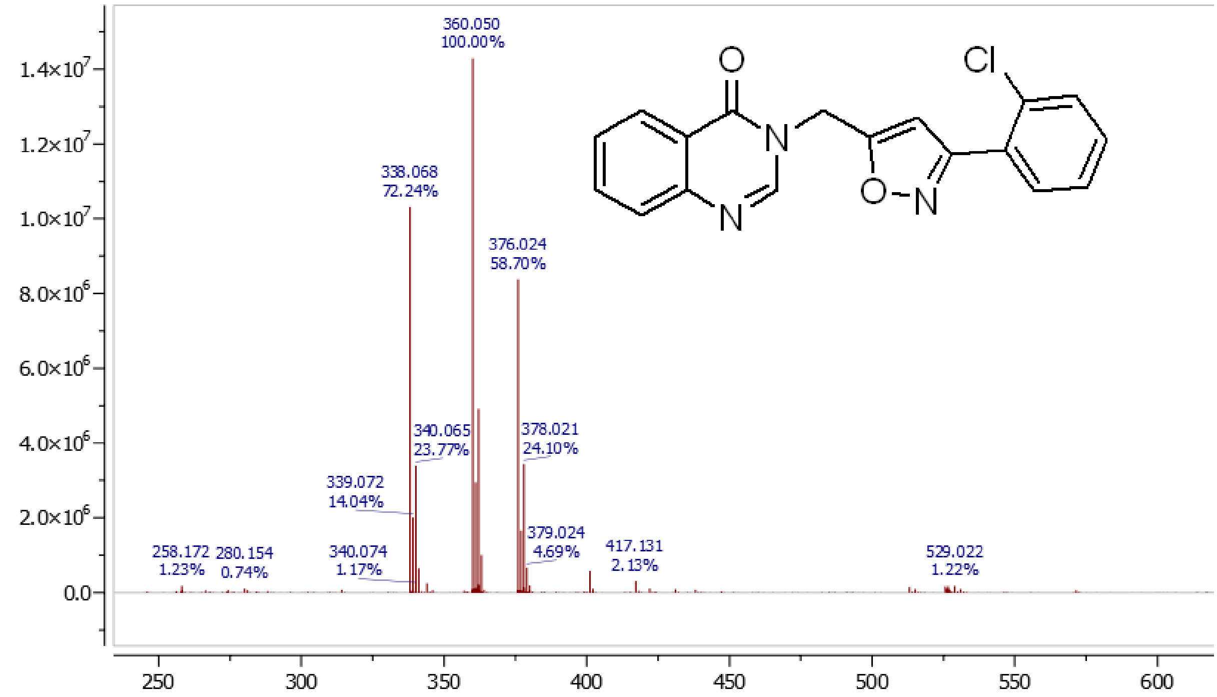

Figure S21. Mass spectrum of compound (**5e**)

References

1. T. Hisano, Recent studies on the modified niementowski 4-quinazolone synthesis. A review, Org Prep Proced Int 5 (1973) 145–193.https://doi.org/10.1080/00304947309355565/ASSET//CMS/ASSET/3A978F86-8829-4E90-8CA9-A2B6345C10BB/00304947309355565.FP.PNG.
